# Zero-determinant strategy in stochastic Stackelberg asymmetric security game

**DOI:** 10.1038/s41598-023-38460-8

**Published:** 2023-07-12

**Authors:** Zhaoyang Cheng, Guanpu Chen, Yiguang Hong

**Affiliations:** 1 Key Laboratory of Systems and Control, Academy of Mathematics and Systems Science, Beijing, 100190 China; 2 School of Mathematical Sciences, University of Chinese Academy of Sciences, Beijing, 100049 China; 3 School of Electrical Engineering and Computer Science, KTH Royal Institute of Technology, 11428 Stockholm, Sweden; 4 Department of Control Science and Engineering, Tongji University, Shanghai, 201804 China; 5 Shanghai Research Institute for Intelligent Autonomous Systems, Tongji University, Shanghai, 210201 China

**Keywords:** Computer science, Applied mathematics

## Abstract

In a stochastic Stackelberg asymmetric security game, the strong Stackelberg equilibrium (SSE) strategy is a popular option for the defender to get the highest utility against an attacker with the best response (BR) strategy. However, the attacker may be a boundedly rational player, who adopts a combination of the BR strategy and a fixed stubborn one. In such a condition, the SSE strategy may not maintain the defensive performance due to the stubbornness. In this paper, we focus on how the defender can adopt the unilateral-control zero-determinate (ZD) strategy to confront the boundedly rational attacker. At first, we verify the existence of ZD strategies for the defender. We then investigate the performance of the defender’s ZD strategy against a boundedly rational attacker, with a comparison of the SSE strategy. Specifically, when the attacker’s strategy is close to the BR strategy, the ZD strategy admits a bounded loss for the defender compared with the SSE strategy. Conversely, when the attacker’s strategy is close to the stubborn strategy, the ZD strategy can bring higher defensive performance for the defender than the SSE strategy does.

Stochastic security games attract more and more attention in many fields such as the cyber-physical system (CPS), the unmanned aerial vehicle (UAV), and the moving target defense (MTD)^1–4^. The stochastic Stackelberg asymmetric security game is one of the important categories to characterize players’ behaviors when the defender faces persistent threats from the attacker. As a fundamental model discussed in the previous works^5,6^, the attacker tends to choose the best response (BR) strategy after observing the defender’ strategy, while the defender aims to maximize its utility considering the attacker. Actually, the defender, as a leader, has an advantage in guiding the attacker’s decision, and the defender picks the optimal strategy based on predicting the attacker’s BR strategy. The corresponding equilibrium is defined as the strong Stackelberg equilibrium (SSE)^5,7,8^.

However, players may not be completely rational due to subjective or objective factors^9,10^ in practice. In fact, a boundedly rational attacker does not strictly adopt the BR strategy. This may result from the limitation of the attacker’s observation, the disturbance of the environment, or the imitative behavior of the attacker. For instance, in MTD problems, the attacker may not directly observe the certain defense strategy because of the disturbance from the administrator (defender)^2,11^. In UAV systems, the malicious UAV may not observe the location or the flight attitude of the legitimate UAV (defender) due to the obstruction in the wild^12^. In CPS, the attacker may design a stealthy attack scheme instead of the BR strategy to avoid the fault detection of the defender^1,13^.

When a boundedly rational attacker loses the ability or interest to achieve the BR strategy, it may turn to a fixed stubborn strategy in most cases. For example, a player prefers a stubborn strategy to avoid being induced to an unsatisfactory outcome in the CPS security^14^ and may choose a fixed credible strategy when the player cannot calculate the BR strategy timely in MTD problems^15^. Against a stubborn attacker, the SSE strategy fails to be regarded as the optimal solution for the defender. In fact, a boundedly rational attacker may choose a mixed strategy, composed by the BR strategy and the stubborn strategy, and such boundedly rational players are common in security problems. For instance, in CPS, the attacker is hesitating between adopting a stubborn strategy or moving as a follower since it needs to consider the failure probability of its own data acquisition system to avoid potential loss^16^. In UAV security problems, a UAV also faces different choices in different stages, since the UAV may lose the location of the defender when going through some complex terrains like in the forest, but fully observes the defender on plains^12^. Thus, various factors, including the potential preference, inherent cognition, and available resources, make the boundedly rational attacker nonnegligible to the defender.

Clearly, due to the stubbornness within a boundedly rational attacker, the original SSE strategy may be no longer suitable for the defender. Thus, it is important to consider other strategies to help the defender maintain its defensive performance. Fortunately, zero-determinate (ZD) strategies provide a powerful idea to unilaterally enforce an advantageous relation between players’ expected utilities, no matter what strategy the opponent selects. Proposed by Press and Dyson^17^ in iterated prisoner’s dilemma (IPD), a ZD strategy means that one player can unilaterally enforce the two players’ expected utilities subjected to a linear relation. Afterward, various ZD strategies have been widely studied to promote cooperation or unilaterally extortion in public goods games (PGG), human-computer interaction (HCI), evolutionary games, etc^18–24^.

Besides, asymmetric matrix games are more realistic than symmetric ones, and there are some challenges to solve the asymmetric games due to the different preferences^25,26^. Currently, there are not many breakthroughs by applying ZD strategies in symmetric games. For example, some works adopt the ZD strategies to persuade the service provider to cooperate in iterated data trading dilemma games^27^ and to deploy as special active defense strategies in IoT devices^28^. Considering the universality and importance of asymmetric games in security, it is necessary to explore the performance of ZD strategies in asymmetric games under security scenarios, since the original analysis of ZD strategies in IPD cannot be directly applied to the asymmetric security game.

In this paper, we are inspired to reveal whether the defender can adopt the ZD strategy against a boundedly rational attacker in stochastic Stackelberg asymmetric security games, in order to make up for SSE strategies’ deficiencies. To this end, we show that the ZD strategy gives a better performance than the SSE strategy does. The main contribution of this work is summarized as follows.

- We apply ZD strategies in asymmetric security games. We first verify the existence of ZD strategies, for the availability of the defender. Besides, against the two special attackers, we investigate the defensive performance of ZD strategies compared with SSE strategies. Specifically, against an attacker with the BR strategy, the ZD strategy admits a bounded loss in the utility compared with the SSE strategy, while against a stubborn attacker, the ZD strategy performs well and brings the defender a higher utility than the SSE strategy does.
- We further analyze a general case where the boundedly rational attacker adopts mixed strategies. When the attacker’s strategy is close to the BR strategy, we provide the defender with appropriate ZD strategies to maintain a bounded loss in defensive performance compared with the SSE strategy, and save the computing resources. Also, when the attacker is close to a stubborn attacker, we show suitable ZD strategies for the defender to get higher defensive performance than SSE strategies.
- We verify our results in two experiments by providing the defender with proper ZD strategies to compare with an SSE strategy^5^. First, we show its performance in MTD problems, where the boundedly rational attacker can directly observe the defender’s strategy and derive its explicit BR strategy^2,11^. Then we show its performance in CPS problems. The setting is more complicated but practical than the considered MTD problems, where the attacker can only observe players’ action history and calculate the BR strategy based on certain mechanisms, like the fictitious play and the Q-learning^7,29^.

## Stochastic Stackelberg asymmetric security game

It is known that, in a stochastic asymmetric security game with the memory of the last stage, an attacker aims to invade two targets and a defender prevents the attack in each stage^2,30^. Consider the stochastic Stackelberg asymmetric security game $\mathcal{G}=\{\mathcal{S},N,\mathcal{D},\mathcal{A},r,P\}$. $\mathcal{S}=\{11,12,21,22\}$ is the set of states, which is composed by the previous attack and defense targets. $N=\{d,a\}$ is the set of players. $\mathcal{D}=\{1,2\}$ and $\mathcal{A}=\{1,2\}$ are the defender’s action set and the attacker’s action set, respectively. $\textbf{r}=\{r_d,r_a\}$ is the reward set of players, where $r_i:\mathcal{D}\times \mathcal{A}\rightarrow \mathbb{R}$, $i\in N$. Besides, $P:\mathcal{S}\times \mathcal{S}\times \mathcal{D}\times \mathcal{A}\rightarrow [0,1]$ is the transition function, where $P(s'|s,d,a)$ shows the probability to the next state $s'\in \mathcal{S}$ from the current state *s* when players take *d*, *a*, and $\sum _{s'\in \mathcal{S}}P(s'|s,d,a)=1$ for $s\in \mathcal{S},d\in \mathcal{D}$, and $a\in \mathcal{A}$.

**Table 1:** Utility matrix.

		Attacker	
		1	2
Defender	1	$(U^d_{11},U^a_{11})$	$(U^d_{12},U^a_{12})$
	2	$(U^d_{21},U^a_{21})$	$(U^d_{22},U^a_{22})$

In this security game, since the state represents the previous players’ actions, $P(s'|s,d,a)=1$ if and only if $s'=(da)$ for any $s\in \mathcal{S}$. Thus, the next state depends on players’ strategies and the current state. For convenience, denote $P(s'|s)$ as the state transition probability to state $s'$ from state *s*, where $s',s\in \mathcal{S}$. Furthermore, in the game $\mathcal{G}$, each player’s strategy depends on the current state, which is also a memory-one strategy. The strategy of the defender is a probability distribution $\pi _d$, where $\pi _d(d|s)\in \Delta \mathcal{D}$ with $\Delta \mathcal{D}$ denoting a probability simplex defined on the space $\mathcal{D}$. Similarly, the strategy of the attacker is $\pi _a$ with $\pi _a(a|s)\in \Delta \mathcal{A}$. Thus, $P(s,s')=\pi _d(d|s)\pi _a(a|s)$, where $s'=(da)$. Set $M=\{P(s|s')\}_{s,s'\in \mathcal{S}}$ as the state transition matrix of this security game. As discussed in previous works^17,31^, we carry forward the investigation with a regular matrix *M*.

At stage *t* in $\mathcal{G}$, each player observes the current state $s_{t}$, and adopts an action according to its strategy. The defender chooses an action $d_t\in \mathcal{D}$, while the attacker chooses an action $a_t\in \mathcal{A}$. The reward of the the defender in stage *t* is denoted by $r_d(d_t,a_t)=U^d_{d_ta_t}$, where $U^d_{d_ta_t}$ is the defender’s utility when the defender protects target $d_t$ and the attacker invades target $a_t$. Similarly, the reward of the attacker in stage *t* is denoted by $r_a(d_t,a_t)=U^a_{d_ta_t}$. The utility martix in each stage is shown Table 1.

### Remark 1

The utility matrix denotes players’ utilities when players choose actions at each stage, based on many typical one-shot security game models^6,32^. For example, consider a critical infrastructure protection problem. If both the defender and the attacker choose target $i\in \{1,2\}$, then the defender, with the utility $U_{ii}^d$, successfully protects the infrastructure under attack, while the attacker, with the utility $U_{ii}^a$, attacks the secure infrastructure with protection. Besides, if the defender chooses target *i* but the attacker chooses target $j \ne i$, then the defender, with the utility $U_{ij}^d$, deploys the protection on the critical infrastructure, while the attacker, with the utility $U_{ij}^a$, invades the vulnerable infrastructure without protection.

The expected long-term utilities in the repeated security game are denoted by

$$\begin{aligned} U_d(\pi _d,\pi _a)&=\mathbb{E}\left( \lim _{T\rightarrow \infty }\sum \limits _{t=0}^T\frac{r_d(d_t,a_t)}{T}\right) ,\\ U_a(\pi _d,\pi _a)&=\mathbb{E}\left( \lim _{T\rightarrow \infty }\sum \limits _{t=0}^T\frac{r_a(d_t,a_t)}{T}\right) , \end{aligned}$$

where $\left\{ d_{t}\sim \pi _d(\cdot |s_{t})\right\} _{t\geqslant 0}$, $\left\{ a_{t}\sim \pi _a(\cdot |s_{t})\right\} _{t\geqslant 0}$, and $\left\{ s_{t}\sim P(\cdot |s_{t-1},d_{t-1},a_{t-1})\right\} _{t>0}$ describe the evolution of states and actions over stage. Additionally, $s_0$ is the initial state ramdomly samplied from $\mathcal{S}$, and the expected utility of each player is the same for any $s_0$, since *M* is convergent. Actually, the expected long-term utility can be transferred into a formulation with determinants. According to Press and Dyson^17^, for $i\in \{d,a\}$, $U_i(\pi _d,\pi _a)=\mathbb{E}\left( \lim \limits _{T\rightarrow \infty }\sum _{t=0}^T\frac{r_d(d_t,a_t)}{T}\right) =\frac{D(\pi _d,\pi _a,S^i)}{D(\pi _d,\pi _a,\textbf{1})},$ where $D(\pi _d,\pi _a,\textbf{f})$ is defined in the Notations of Supplementary Information, for $\textbf{f}=[f_1,f_2,f_3,f_4]^T\in \mathbb{R}^4$, and $S^i=[U^i_{11},U^i_{12},U^i_{21},U^i_{22}]$.

### Assumption 1

The utilities satisfy $\min \{U_{11}^d,U_{22}^d\}>\max \{U_{12}^d,U_{21}^d\}$. Moreover, $U_{11}^a<U_{12}^a$, and $U_{22}^a<U_{21}^a$.

Different from IPD^17,33^, the asymmetry in this security game comes from the actual security mechanism. Specifically, the defender tends to resist attacks, that is, to protect the vulnerable target, and the attacker tends to implement invasions on the unprotected target. The above represents a wide class of asymmetric game in security scenarios, which is summarized as Assumption 1. Similar investigations have been broadly discussed in the literature of various security games^6,32,34^.

## Boundedly rational attacker

In the stochastic Stackelberg asymmetric security game, the defender is a leader and declares a strategy in advance, while the attacker is a follower and chooses its strategy after observing the defender’s strategy. In most cases, the attacker may choose the BR strategy when it obtains the defender’s strategy.

After observing the defender’s strategy $\pi _d$, the attacker may choose the BR strategy^7^ as follows:

$$\begin{aligned} \pi _a^{BR}(\pi _d)\in \textbf{BR}(\pi _d)=\mathop {\text{argmax}}\limits _{\pi _a\in \Delta \mathcal{B}} U_a(\pi _d,\pi _a). \end{aligned}$$

Without loss of generality, the follower can break ties optimally for the leader if there are multiple options. In this case, the defender aims to maximize its utility considering the attacker, and the equilibrium is defined as the strong Stackelberg equilibrium (SSE)^5,7,8,35^.

### Definition 1

A strategy profile $(\pi _d^{SSE},\pi _a^{SSE})$ is said to be a SSE of $\mathcal{G}$ if

$$\begin{aligned}&(\pi _d^{SSE}, \pi _a^{SSE})\in \mathop {argmax }\limits _{\pi _d, \pi _a\in {{\textbf{BR }}}({\pi _d})}U_d(\pi _d,\pi _a). \end{aligned}$$

When the attacker chooses the BR strategy after observing the defender’s strategy, the SSE strategy $\pi _d^{SSE}$ is optimal for the defender, and the defender has an advantage in guiding the attacker’s strategy decision. Besides, if the attacker only observes players’ action history instead of the defender’s strategy directly, the attacker can also choose the BR strategy by some methods such as the fictitious play and the Q-learning method^7,29^.

However, the attacker may not always choose the BR strategy in security problems, due to subjective or objective factors such as the limitation of the attacker’s observation, the disturbance of the environment, and the imitative behavior of the attacker^1,2,11^. In practice, the attacker may turn to other strategies. A fixed stubborn strategy, which is not influenced by the defender, is one of the most likely options for the attacker due to its potential preference, inherent cognition, and available resources^14,15^.

Denote the stubborn strategy in this security game by $\pi _a^*$, while the corresponding attacker is actually a stubborn player. In fact, the attacker intends to keep its action once it finds the most attractive target. For instance, in MTD, there always exists the most vulnerable target for the hacker, and the hacker has no intention to change its attack target once it finds the target^36^. Besides, a UAV tends to keep attacking the current optimum target when it has a limited vision and lacks resources to detect others^37^. Without loss of generality, we consider that there exists a target which is more attractive than the other for the attacker, and summarize the above in the following assumption, which was also broadly considered in previous works^14,36,37^.

### Assumption 2

Target 1 is more attractive than target 2 for the attacker, i.e., $\pi _a^*(1|11)=\pi _a^*(1|21)=1$.

In fact, either the BR strategy or the stubborn strategy may not be the single optimal option for the attacker. The attacker may adopt a mixed strategy composed by both strategies. The attacker, in this case, is actually called a boundedly rational player, and is not unusual in reality. For instance, the attacker may be hesitating between BR strategies and stubborn strategies due to the errors of the data acquisition system in MTD, and data missing in UAV^12,16,38^. Thus, we formulate the mixed strategy as follows.

### Definition 2

The attacker’s strategy $\pi _a^{\lambda }(\pi _d,\pi _a^*)$ is called a boundedly rational strategy if

$$\begin{aligned} \pi _a^{\lambda }(\pi _d,\pi _a^*)=\lambda \pi _a^{BR}(\pi _d) + (1-\lambda ) \pi _a^*, \lambda \in [0,1]. \end{aligned}$$

For the defender’s strategy $\pi _d$, we consider that the boundedly rational attacker adopts the BR strategy $\pi _a^{BR}(\pi _d)\in \textbf{BR}(\pi _d)$ with probability $\lambda$ and the stubborn strategy $\pi _a^*$ with probability $1-\lambda$^10,39^. Therefore, when the attacker selects the stubborn strategy, the defender loses the advantage in guiding the attacker’s strategy decision, and the SSE strategy may not maintain the defensive performance due to the stubbornness therein. Thus, the SSE strategy is no longer suitable for the defender against a boundedly rational attacker. It is important to study other strategies to help the defender maintain its defensive performance.

## Performance of ZD strategy

In this section, we introduce the ZD strategy for the defender in the stochastic Stackelberg asymmetric security game. At first, we show the definition of the ZD strategy for the defender and analyze the existence of the ZD strategy. Besides, we explore the performance of the ZD strategy compared with the SSE strategy. Also, we present proofs of our Theorems in the Supplementary Information.

### ZD strategy for the defender

Proposed by Press and Dyson^17^, ZD strategies mean that one player can unilaterally enforce the two players’ expected utilities subjected to a linear relation, which have been widely studied to promote cooperation or unilaterally extortion in PGG, HCI, and evolutionary games^19–21^. For this stochastic Stackelberg asymmetric security game *G*, the defender’s ZD strategy^17,33,40^ is defined as follows:

#### Definition 3

The defender’s strategy $\pi _d^{ZD}$ is called a ZD strategy if

1 $$\begin{aligned} \pi _d^{ZD}(1)&=\eta \textbf{S}^{d} +\beta \textbf{S}^a +\gamma \textbf{1}_4+\hat{\pi },\\ \pi _d^{ZD}(2)&=1-\pi ^{ZD}_d(1), \end{aligned}$$

where $$\eta ,\beta ,\gamma \in \mathbb{R}$$ and $$\hat{\pi }=[1,1,0,0]^T$$.

Let $\pi _d^{ZD}(k)=[\pi _d(k|11),\pi _d(k|12),\pi _d(k|21),\pi _d(k|22)]^T,$ $k\in \{1,2\}$. The defender’s all feasible ZD strategies are denoted as the following set

$$\begin{aligned} \Xi =\left\{ \pi ^{ZD}_d\in \Delta \mathcal{D}|\pi ^{ZD}_d(1)=\eta \textbf{S}^{d} +\beta \textbf{S}^a +\gamma \textbf{1}_4+\hat{\pi }, \pi _d^{ZD}(2)=1-\pi _d^{ZD}(1),\eta ,\beta ,\gamma \in \mathbb{R} \right\} . \end{aligned}$$

It is called zero-determinant (ZD) that, if the defender adopts the ZD strategy with (1), then players’ expected utilities are subjected to a linear relation:

$$\eta U_d(\pi _d^{ZD},\pi _a)+\beta U_a(\pi _d^{ZD},\pi _a)+\gamma =0, \ \forall \pi _a\in \Delta \mathcal{B}.$$

Take $\pi ^{ZD}(\eta ,\beta ,\gamma )$ as the corresponding ZD strategy. With the help of the ZD strategy’s unilateral enforcement in players’ utilities, we aim to investigate whether the defender can adopt the ZD strategy to better maintain its defensive performance than the original SSE strategy against a boundedly rational attacker.

In what follows, we investigate the existence of ZD strategies to guarantee the availability for the defender.

### Existence of ZD strategy

Actually, the ZD strategy cannot enforce an arbitrary linear relation between two players’ utilities since it must belong to the implementer’s strategy set. Thus, a feasible linear relation enforced by ZD strategies is fundamental for further analysis. The following lemma provides a necessary and sufficient condition for the feasibility of a linear relationship between players’ utilities.

#### Lemma 1

Under Assumption 1, there exists a ZD strategy which enforces $\eta U_d+\beta U_a+\gamma =0$ if and only if either of the following two inequalities is satisfied.

2 $$\begin{aligned}{} & {} \max \limits _{s\in \{11,12\}} \eta U_{s}^d +\beta U_{s}^a \leqslant -\gamma \leqslant \min \limits _{s\in \{21,22\}} \eta U_{s}^d+\beta U_{s}^a. \end{aligned}$$

2 $$\begin{aligned}{} & {} \max \limits _{s\in \{21,22\}} \eta U_{s}^d+\beta U_{s}^a \leqslant -\gamma \leqslant \min \limits _{s\in \{11,12\}} \eta U_{s}^d +\beta U_{s}^a. \end{aligned}$$

Lemma 1 implies that the defender can adopt the ZD strategy $\pi ^{ZD}(\eta ,\beta ,\gamma )$, where $\eta$, $\beta$, and $\gamma$ satisfy (2) or (3), to enforce an ideal linear relation between players’ excepted utilities. Actually, Lemma 1 extends the application of ZD strategies since the payoff matrix in security games is not as symmetric as that in IPD games^41^. Moreover, Lemma 1 covers the following cases.

- The defender can unilaterally restrict attacker’s utility if $U_{11}^a>U_{12}^a$. If the defender takes $\pi ^{ZD}(\eta ,\beta ,\gamma )$ with $\eta =0, \beta \ne 0$, and $U_{12}^a \leqslant -\frac{\gamma }{\beta } \leqslant U_{11}^a$, then the defender ZD can unilaterally restrict the attacker’s utility as $-\frac{\gamma }{\beta }\in [U_{11}^a,U_{12}^a]$, which is the same as the equalizer strategy in IPD games^17,18^.
- The defender can unilaterally restrict its own utility if $U_{12}^d>U_{22}^d$. If the defender takes $\pi ^{ZD}(\eta ,\beta ,\gamma )$ with $\eta \ne 0, \beta =0$, and $U_{22}^d \leqslant -\frac{\gamma }{\eta }\leqslant U_{12}^d$, then the defender can unilaterally restrict the defender’s utility between $U_{22}^d$ and $U_{12}^d$, which is consistent with the result in MTD problems^28^.

Based on the existence condition of feasible linear relations enforced by ZD strategies, we can further analyze whether there exists at least one ZD strategy in the security game $\mathcal{G}$. For simplification, for any $x_1,x_2,y_1,y_2\in \mathbb{R}$ with $x_1\ne x_2$, denote

$$\begin{aligned} \Gamma ^{-}(x_1,y_1,x_2,y_2)=\left\{ (x,y)|y-y_1\leqslant \frac{y_2-y_1}{x_2-x_1} (x-x_1)\right\} ,\\ \Gamma ^{+}(x_1,y_1,x_2,y_2)=\left\{ (x,y)|y-y_1\geqslant \frac{y_2-y_1}{x_2-x_1} (x-x_1)\right\} . \end{aligned}$$

Actually, $\Gamma ^{-}$ ($\Gamma ^{+}$) is the region above (below) the line going through points $(x_1,y_1)$ and $(x_2,y_2)$. Then the following theorem shows a sufficient condition for the existence of a ZD strategy in $\mathcal{G}$.

#### Theorem 1

Under Assumption 1, there exists at least one ZD strategy of the defender in $\mathcal{G}$ if either of the following two relations is satisfied.

$$\begin{aligned} (U_{21}^d,U_{21}^a) ,(U_{22}^d,U_{22}^a) \in \Gamma ^{+}(U_{11}^d,U_{11}^a,U_{12}^d,U_{12}^a).\\ (U_{21}^d,U_{21}^a) ,(U_{22}^d,U_{22}^a) \in \Gamma ^{-}(U_{11}^d,U_{11}^a,U_{12}^d,U_{12}^a). \end{aligned}$$

**Figure 1:**
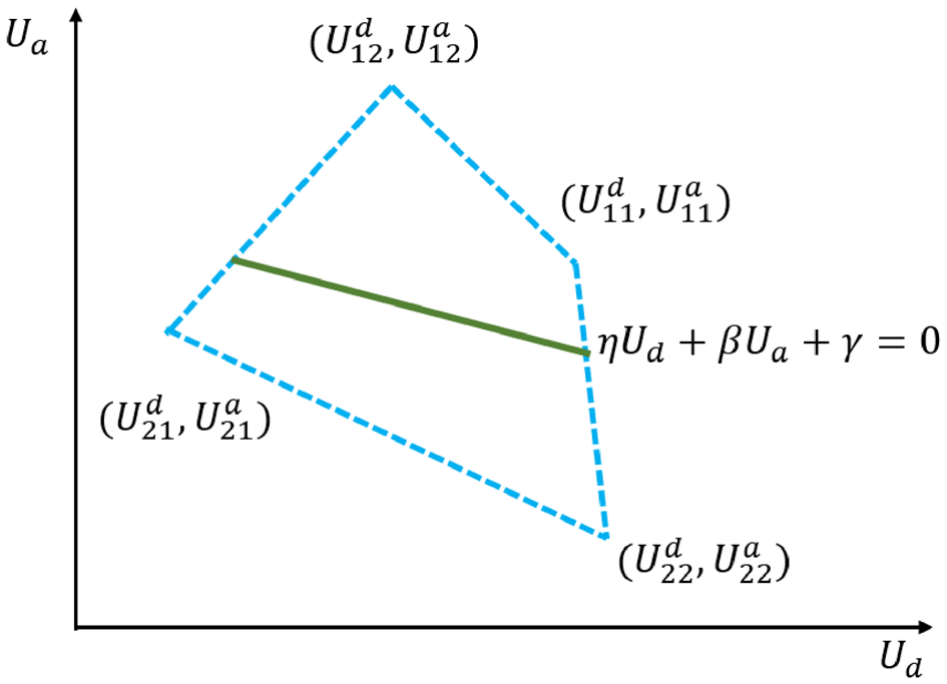
$(U_{11}^d,U_{11}^a)$ and $(U_{12}^d,U_{12}^a)$ lie in the one side of the line $\eta U_a +\beta U_b+\gamma =0$, while $(U_{21}^d,U_{21}^a)$ and $(U_{22}^d,U_{22}^a)$ lie in the other side.

Theorem 1 shows that, in the security game $\mathcal{G}$, the defender is able to choose a ZD strategy once $(U_{21}^d,U_{21}^a)$ and $(U_{22}^d,U_{22}^a)$ lie in the same side of the line going through points $(U_{11}^d,U_{11}^a)$ and $(U_{12}^d,U_{12}^a)$, as shown in Fig. 1. Hence, in this paper, we focus on the situation where there exists at least a ZD strategy, since selecting ZD strategies for the defender is based on its existence. In fact, the two conditions are interchangeable. For example, if $(U_{21}^d,U_{21}^a) ,(U_{22}^d,U_{22}^a) \in \Gamma ^{-}(U_{11}^d,U_{11}^a,U_{12}^d,U_{12}^a)$, then we can get $(U_{21}^d,U_{21}^a),(U_{22}^d,U_{22}^a) \in \Gamma ^{-}(U_{11}^d,U_{11}^a,U_{12}^d,U_{12}^a)$ by swaping the values of $(U_{11}^d,U_{11}^a)$ and $(U_{22}^d,U_{22}^a)$, and swaping the values of $(U_{12}^d,U_{12}^a)$ and $(U_{21}^d,U_{21}^a)$. Hence, without loss of generality, the following results in this paper are established with the condition $(U_{21}^d,U_{21}^a),(U_{22}^d,U_{22}^a) \in \Gamma ^{-}(U_{11}^d,U_{11}^a,U_{12}^d,U_{12}^a)$.

### ZD strategy in two special cases

For understanding easily, we start with two special cases: $\lambda =1$, where the attacker takes BR strategies^7^, and $\lambda =0$, where the attacker is a stubborn player with stubborn strategies^14,15^.

**When** $\varvec{\lambda =1}$, the attacker chooses ${\textbf{BR}}(\pi _d)$ after observing the defender’s strategy $\pi _d$. Recall the definition of $(\pi _d^{SSE},\pi _a^{SSE})$ as a SSE and $U_d^{SSE}$ as the defender’s utility from an SSE strategy.

#### Lemma 2

Under Assumption 1, for any $\pi _d^{ZD}\in \Xi$ and $\pi _a\in BR(\pi _d^{ZD})$, $U_d(\pi _d^{ZD},\pi _a)\leqslant U_d(\pi _d^{SSE},\pi _a^{SSE})$.

Lemma 2 reveals that the SSE strategy always brings the highest utility for the defender when facing an attacker with the BR strategy. In this case, the upper limit of the performance of ZD strategies cannot surpass the defender’s utility an SSE strategy. In spite of this, the following theorem tells that ZD strategies admit a bounded loss compared with SSE strategies.

#### Theorem 2

Under Assumption 1,

$$\begin{aligned}&\min \limits _{\pi _d^{ZD}\in \Xi }U_d\left( \pi _d^{SSE}, \pi _a^{BR}(\pi _d^{SSE})\right) \!-\!U_d\left( \pi _d^{ZD}, \pi _a^{BR}(\pi _d^{ZD})\right) \\&\quad \quad = \left\{ \begin{array}{ll} 0, &{}\quad \quad \quad \text{if } U_{11}^d\geqslant U_{21}^d,\\ U_d^{SSE}-U_{12}^d, &{}\quad \quad \quad \text{if }U_{11}^d < U_{21}^d. \end{array} \right. \end{aligned}$$

The corresponding ZD strategy is

$$\begin{aligned} \pi _d^{ZD}=\! \!\left\{ \begin{array}{ll} \pi ^{ZD}\!(\!-\!k_1,\! 1,\! k_1U_{11}^d \!\!-\! U_{11}^a\!), &{}\quad \text{if} \ U_{11}^d \!\geqslant \! U_{22}^d, \ U_{11}^a\!\geqslant \! U_{21}^a,\\ \pi ^{ZD}(0,\!1,\!-U_{21}^a), &{}\quad \text{if} \ U_{11}^d\!< \!U_{22}^d,\ U_{11}^a\!\geqslant \! U_{21}^a,\\ \pi ^{ZD}\!(\!-\!k_2,\!1,\! k_2 U_{12}^d\!\!-\!U_{12}^a\!), &{}\quad \text{otherwise,} \end{array} \right. \end{aligned}$$

where $$0\leqslant k_1\leqslant \frac{U_{11}^a-U_{21}^a}{U_{11}^d-U_{21}^d}$$, and $$\frac{U_{22}^a-U_{12}^a}{U_{22}^d-U_{12}^d} \leqslant k_2\leqslant \frac{U_{11}^a-U_{12}^a}{U_{11}^d-U_{12}^d}$$.

Theorem 2 shows that the defender can adopt ZD strategies to get a bounded loss in the utility compared with SSE strategies. On the one hand, when $U_{11}^a\geqslant U_{21}^a$, the ZD strategy in Theorem 2 is an SSE strategy and brings the defender the same utility as SSE strategies. On the other hand, if the defender can endure the bounded loss, then adopting the corresponding ZD strategy is also a good choice to avoid the complicated calculation for SSE strategies, since deriving SSE strategies needs solve a bi-level optimization problem.

**When** $\varvec{\lambda =0}$, the attacker chooses the stubborn strategy $\pi _a^*$. The SSE strategy may not bring the defender a high utility, since the stubborn attacker does not choose the BR strategy as the defender’s expectation. In this case, due to the ZD strategy’s unilateral enforcement in players’ utilities, a ZD strategy can exactly play an essential role to enforce desired utilities for the defender and even to bring a higher utility than the original SSE strategy does.

#### Theorem 3

Under Assumptions 1 and 2, there exists a ZD strategy $\pi _d^{ZD}= \pi ^{ZD}(-k, 1, kU_{12}^d -U_{12}^a)$ with $k=\frac{U_{11}^a-U_{12}^a}{U_{11}^d-U_{12}^d}$ such that

$$\begin{aligned} U_d(\pi _d^{ZD},\pi _a^*)\geqslant U_d(\pi _d^{SSE},\pi _a^*). \end{aligned}$$

In fact, the ZD strategy $\pi ^{ZD}(-k, 1, kU_{12}^d -U_{12}^a)$ enforces the linear relation between players’ expected utilities going through $(U_{12}^a,U_{12}^b)$ and $(U_{11}^d,U_{11}^a)$. The infimum of the ZD strategy $\pi ^{ZD}(-k, 1, kU_{12}^d -U_{12}^a)$ is not lower than that of any other ZD strategy, including the ones which can unilaterally set the defender’s utility^28^. Moreover, according to its proof, when facing the stubborn attacker, this ZD strategy $\pi _d^{ZD}$ brings an increase $U_{11}^d-\frac{U_{11}^d \pi _d^{SSE}(1|21)+U_{21}^d\pi _d^{SSE}(2|11)}{\pi _d^{SSE}(2|11)+\pi _d^{SSE}(1|21)}$ in utility for the defender compared with the SSE strategy.

### ZD strategy in general case

It is time to consider the general case **when** $\varvec{\lambda \in [0,1]}$. Here, the boundedly rational attacker chooses the BR strategy with probability $\lambda$ and the stubborn strategy $\pi _a^*$ with probability $1-\lambda$, i.e., $\pi _a^{\lambda }(\pi _d,\pi _a^*)$ in Definition 2. For convenience, take

2 $$\begin{aligned}{} &\Gamma _1=\{\lambda \in [0,1]|(U_{d}^{SSE}-U_{12}^d)D({\textbf{1}})\lambda ^4- A(1-\lambda )^4-B\lambda (1-\lambda )\geqslant 0\},\\&\Gamma _2=\{\lambda \in [0,1]|(U_{12}^d-U_d^{SSE})D({\textbf{1}})\lambda ^4+ A(1-\lambda )^4-B\lambda (1-\lambda )\geqslant 0\}, \end{aligned}$$

3 $$\begin{aligned}{} & {} H(\pi _d^{ZD},\pi _d^{SSE},\pi _a^*,\lambda )=\frac{\left( U_d^{SSE}-U_{12}^d\right) C(\pi _d^{ZD},\pi _d^{SSE},\pi _a^*,1)\lambda ^4 - A(1-\lambda )^4+B\lambda (1-\lambda )}{C(\pi _d^{ZD},\pi _d^{SSE},\pi _a^*,\lambda )}, \end{aligned}$$

where the parameters therein are shown in the Notations of the Supplementary Information. Intuitively, the ZD strategy may bring the defender a similar performance as shown in Theorem 2 when $\lambda$ is close to 1, and a similar performence as given in Theorem 3 when $\lambda$ is close to 0. In fact, one main result of this subsection is given in the following theorem.

#### Theorem 4

Under Assumptions 1 and 2, for $\lambda \in \Gamma _1$ in (4), there is

$$\begin{aligned}&\min \limits _{\pi _d^{ZD}\in \Xi } U_d\left( \pi _d^{SSE},\pi _a^{\lambda }(\pi _d^{SSE},\pi _a^*)\right) - U_d\left( \pi _d^{ZD},\pi _a^{\lambda }(\pi _d^{ZD},\pi _a^*)\right) \\&\quad \quad \leqslant \left\{ \begin{array}{ll} 0, &{} \text{if} \ U_{11}^d\!\geqslant \! U_{22}^d, \ U_{11}^a\!\geqslant \!U_{21}^a, \\ H(\pi _d^{ZD},\pi _d^{SSE},\pi _a^*,\lambda ), &{} \text{otherwise}, \end{array} \right. \end{aligned}$$

where $H(\pi _d^{ZD},\pi _d^{SSE},\pi _a^*,\lambda )$ was defined in (5). The corresponding ZD strategy is

$$\begin{aligned} \pi _d^{ZD}=\!\!\left\{ \! \begin{array}{ll} \!\pi ^{ZD}(-k_1,\! 1,\! k_1U_{11}^d \!-\!U_{11}^a), &{} \text{if} \ U_{11}^d\! \geqslant \!U_{22}^d, \ U_{11}^a\!\geqslant \!U_{21}^a,\\ \!\pi ^{ZD}(-k_2,\!1, \!k_2 U_{12}^d\!-\!U_{12}^a), &{}\text{otherwise}, \end{array} \right. \end{aligned}$$

where $$0\leqslant k_1\leqslant \frac{U_{11}^a-U_{21}^a}{U_{11}^d-U_{21}^d}$$, and $$k_2=\frac{U_{11}^a-U_{12}^a}{U_{11}^d-U_{12}^d}$$.

Theorem 4 provides the set $\Gamma _1$ for the defender, in which the ZD strategy brings a bounded loss in defensive performance, even though the ZD strategy cannot surplus the SSE strategy. Thus, for the boundedly rational attacker with $\lambda \in \Gamma _1$, if the defender does not care too much about losing a little utility, then the defender can adopt the corresponding ZD strategy since adopting the ZD strategy in Theorem 4 avoids paying vast resources to solve a bi-level optimization problem for the SSE strategy. Moreover, if $U_{11}^a\geqslant U_{21}^a$ and $U_{11}^d\geqslant U_{22}^d$, the ZD strategy can bring the defender the same utility as an SSE strategy, which means that the defender can still adopt ZD strategies.

Although $\Gamma _1$ seems complicated to verify, we can easily check some typical cases of $\lambda$ when they belong to $\Gamma _1$. For instance, $\lambda =1$ is always in $\Gamma _1$. In this case, the attacker tends to take the BR strategy, which is consistent with Theorem 2. Actually, $\lambda$ is in $\Gamma _1$ if $\lambda$ is close to 1, which means that the attacker tends to choose the BR strategy. Also, we provide a subset of $\Gamma _1$, which can be verified easily by the defender.

#### Corollary 1

Under Assumptions 1 and 2, if $\lambda \in [\frac{1}{2},1]$ and

$$\begin{aligned}&4(U_{11}^d-U_{21}^d)\pi _{d}^{SSE}(2|11)(1-\lambda )^4\\ \leqslant& \left( \frac{1}{4}(U_d^{SSE}-U_{12}^d)D({\textbf{1}})-B \right) \left( \pi _d^{SSE}(2|11)+\pi _d^{SSE}(1|21)\right) , \end{aligned}$$

then

$$\begin{aligned}&\min \limits _{\pi _d^{ZD}\in \Xi } U_d\left( \pi _d^{SSE},\pi _a^{\lambda }(\pi _d^{SSE},\pi _a^*)\right) - U_d\left( \pi _d^{ZD},\pi _a^{\lambda }(\pi _d^{ZD},\pi _a^*)\right) \\&\leqslant \left\{ \begin{array}{ll} 0, &{} \text{if} \ U_{11}^a\geqslant U_{21}^a, \ U_{11}^d\geqslant U_{22}^d,\\ H(\pi _d^{ZD},\pi _d^{SSE},\pi _a^*,\lambda ), &{} \text{otherwise}. \end{array} \right. \end{aligned}$$

At last, we analogously consider the situation when $\lambda$ is close to 0, i.e., the attacker tends to take the stubborn strategy in the following theorem.

#### Theorem 5

Under Assumptions 1 and 2, if $\lambda \in \Gamma _2$ in (4), then there exists a ZD strategy $\pi _d^{ZD}=\pi ^{ZD}(-k, 1, k U_{21}^a -U_{21}^b)$ such that

$$\begin{aligned} U_d(\pi _d^{ZD},\pi _a^{\lambda }(\pi _d^{ZD},\pi _a^*))\!\geqslant \!U_d(\pi _d^{SSE},\pi _a^{\lambda }(\pi _d^{SSE},\pi _a^*)), \end{aligned}$$

where $$k=\frac{U_{11}^b-U_{12}^b}{U_{11}^a-U_{12}^a}.$$

Theorem 5 provides the set $\Gamma _2$ for the defender to adopt the ZD strategy to get a higher utility than the original SSE strategy. Notice that the corresponding ZD strategy yields wonderful performance. If $\lambda \in \Gamma _2$, the defender can confidently select the corresponding ZD strategy, since the ZD strategy brings the defender higher defensive performance than an SSE strategy does.

Clearly, $\lambda =0$ is always in $\Gamma _2$, which is consistent with the results in Theorem 3. Actually, $\lambda$ is in $\Gamma _2$ if $\lambda$ is close to 0, which means that the attacker tends to be a stubborn attacker. Also, we provide a subset of $\Gamma _2$ for the defender, which can be verified easily by the defender.

#### Corollary 2

Under Assumptions 1 and 2, if $\lambda \in [0,\frac{1}{2}]$ and

$$\begin{aligned} 4(U_d^{SSE}-U_{12}^a)D({\textbf{1}})\lambda ^4\leqslant \frac{1}{4}A-B, \end{aligned}$$

then there exists $\pi _d^{ZD}\in \Xi$ such that

$$\begin{aligned} U_d(\pi _d^{ZD},\pi _a^{\lambda }(\pi _d^{ZD},\pi _a^*))\!\geqslant \!U_d(\pi _d^{SSE},\pi _a^{\lambda }(\pi _d^{SSE},\pi _a^*)). \end{aligned}$$

**Figure 2:**
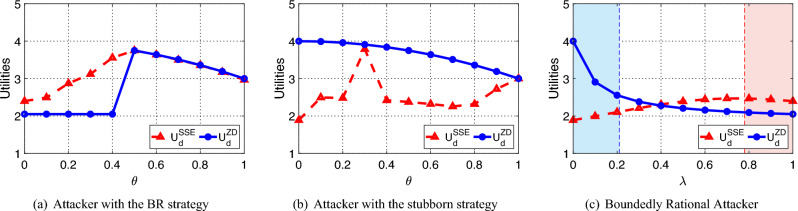
Performance of the ZD strategy compared with the SSE strategy in MTD problems. Red dotted lines describe the defender’s expected utilities with an SSE strategy, while blue solid lines describe them with the corresponding ZD strategy. In (**c**), the red (blue) region shows the bound of $\Gamma _1$ ($\Gamma _2$) according to Theorem 4 (Theorem 5).

## Applications

For illustration, we provide experiments to verify that the ZD strategy can help the defender maintain its defensive performance against a boundedly rational attacker, where the baseline is the original SSE strategy.

**Table 2:** Utility matrix in MTD problems.

		Attacker	
		1	2
Defender	1	$(R^d_{11}\!-\!\frac{Y_2}{2},\!R^a_{11}\!-\!C_1)$	$(R^d_{12}\!-\!\frac{Y_2}{2},R^a_{12}\!-\!C_2)$
	2	$(R^d_{21}\!-\!\frac{Y_1}{2},\!R^a_{21}\!-\!C_1)$	$(R^d_{22}\!-\!\frac{Y_1}{2},\!R^a_{22}\!-\!C_2)$

### In MTD problems

Let us consider an MTD problem, where the attacker can directly observe the defender’s strategy and take its explicit BR strategy^2,11^. Take $Y_i$ as the cost of the defender moving the defense resource from target *i* to the other target, and take $C_i$ as the cost of the attacker invading target *i*. Similar to^28^, we also use the average to approximate the transfer cost. Also, take $R^d_i$ and $R^a_i$ as the reward and loss for two players in the state $i\in \mathcal{S}$. Thus, the utility matrix in MTD problems is shown in Table 2. Take $R_s^d=d_s^1\theta +d_s^0$, and $R_s^a=a_s^1\theta +a_s^0$ for any $s\in \mathcal{S}$, where $\theta \in [0,1]$, and $d_s^k$ and $a_s^k$ are parameters, which describe different situations, respectively, for any $s\in \mathcal{S}$ and $k\in \{1,2\}$.

As shown in Fig. 2a, for an attacker with the BR strategy, the expected utility of the defender with an SSE strategy is always higher than that with a ZD strategy. Besides, in Fig. 2b, for an attacker with the stubborn strategy, the expected utility of the defender with adopting the ZD strategy in Theorem 3 is always higher than that with adopting an SSE strategy. Moreover, in Fig. 2c, $U_d^{ZD}$ is always higher than $U_d^{SSE}$ when $\lambda <0.21$, which is consistent with Theorem 5. Also, $U_d^{SSE}$ is always higher than $U_d^{ZD}$ when $\lambda >0.78$, which is consistent with Theorem 4, and the difference between the two utilities is bounded.

**Figure 3:**
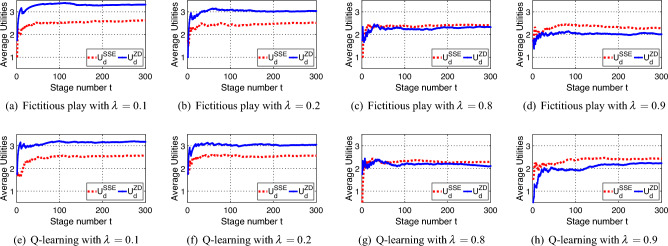
Performance of the ZD strategy compared with the SSE strategy in CPS with different mechanisms. Red dotted lines show the defender’s average utility with an SSE strategy, while blue solid lines show the defender’s average utility with the corresponding ZD strategy in Theorem 4 or Theorem 5.

### In CPS problems

Here we consider a CPS problem with a defender as a system administrator and an attacker as a jammer or an eavesdropper. Here, different from the previous experiment, the attacker can only observe players’ action history without directly receiving the defender’s strategy. The attacker adopts the BR strategy based on the fictitious play^29^ or the Q-learning method^7^, whose details are provided in Supplementary Information.

**Figure 4:**
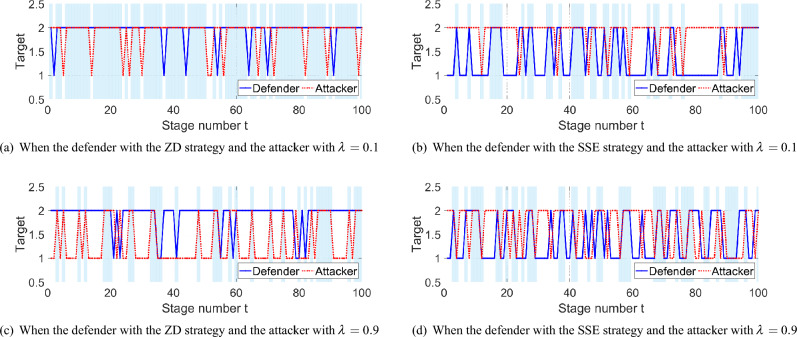
Target interaction between the defender and the attacker. Blue solid lines show the target that the defender protects, while red dotted lines show the target that the attacker attacks in each stage. The blue region shows that the defender and the attacker choose the same target.

As shown in Fig. 3a–f, when $\lambda$ is close to 0, like 0.1 in Fig. 3a,e, and 0.2 in Fig. 3b,f, the average utility of the defender with the ZD strategy is higher than that with the SSE strategy. Besides, when $\lambda$ is close to 1, like 0.8 in Fig. 3c,g, and 0.9 in Fig. 3d,h, although the average utility with the ZD strategy is lower than that with the SSE strategy, the loss is small enough for the defender. Moreover, as shown in Fig. 4a–d, we present the switching process of target interactions between the defender and the attacker. Specifically, when the two players choose the same target, the defender successfully protects the right target and gains a high utility, as shown in the blue region of Fig. 4. When $\lambda =0.1$ and the attacker tends to be a stubborn one, the defender chooses the ZD strategy and the SSE strategy in Fig. 4a,b, respectively. Notice that there are more blue regions in Fig. 4a than that in Fig. 4b. Thus, the defender with the ZD strategy succeeds in protecting more frequently than that with the SSE strategy. It is consistent with Theorem 5 that the ZD strategy has a better performance than the SSE strategy for the defender when the attacker tends to be stubborn. Similarly, consider $\lambda =0.9$ in Fig. 4c,d. The defender with the ZD strategy still has a good performance in protection, compared with the defender with the SSE strategy, when the attacker tends to choose the BR strategy, which is consistent with Theorem 4. Thus, the defender can also adopt ZD strategies to maintain its defensive performance with a bounded and tolerable loss, and avoid the complicated computing in SSE strategies.

## Discussion

We have focused on stochastic Stackelberg asymmetric security games in this paper. Due to the stubbornness within the boundedly rational attacker, we have investigated the defensive performance of ZD strategies, and have analyzed whether the ZD strategies can make up for the deficiencies of SSE strategies in such circumstances. Also, we have provided experiments to support our methodology by employing proper ZD strategies for the defender.

Actually, our results can be extended to some security problems with multiple targets. For example, consider the case that the targets in the security game can be divided into two categories. Each player’s utilities are the same when choosing any two targets belonging to one category, while the player’s utilities are different when choosing any two targets belonging to different categories. In such a situation, the defender can still adopt similar ZD strategies in this paper to improve its defensive performance.

Indeed, in multi-target asymmetric stochastic security games, there are still many challenges in applying the ZD strategy against boundedly rational attackers. For example, in multi-target settings, the expected utility of the defender will be composed of complex polynomials in $\lambda$, and each polynomial is the determinant of a $(n^2 \times n^2)$ matrix with a very high degree. Applying ZD strategies in general multi-target security games is still an open problem and deserves more exploration.

### Supplementary Information

Supplementary Information.

## Data Availability

The datasets used and analysed during the current study available from the corresponding author on reasonable request.

## References

[1] Xiao, K. *et al.* Dynamic defense strategy against stealth malware propagation in cyber-physical systems. In *IEEE INFOCOM 2018: IEEE Conference on Computer Communications*, 1790–1798 (2018).

[2] Feng, X., Zheng, Z., Cansever, D., Swami, A. & Mohapatra, P. A signaling game model for moving target defense. In *IEEE INFOCOM 2017-IEEE Conference on Computer Communications*, 1–9 (2017).

[3] Kovtun V, Izonin I, Gregus M (2022). Reliability model of the security subsystem countering to the impact of typed cyber-physical attacks. Sci. Rep..

[4] Wu Z, Pan L, Yu M, Liu J, Mei D (2022). A game-based approach for designing a collaborative evolution mechanism for unmanned swarms on community networks. Sci. Rep..

[5] Vorobeychik, Y. & Singh, S. Computing Stackelberg equilibria in discounted stochastic games. In *Proceedings of the AAAI Conference on Artificial Intelligence* 26, 1478–1484 (2012).

[6] Korzhyk D, Yin Z, Kiekintveld C, Conitzer V, Tambe M (2011). Stackelberg vs. Nash in security games: An extended investigation of interchangeability, equivalence, and uniqueness. J. Artif. Intell. Res..

[7] Li, K. & Hao, D. Cooperation enforcement and collusion resistance in repeated public goods games. In *Proceedings of the AAAI Conference on Artificial Intelligence* 33, 2085–2092 (2019).

[8] Cheng Z, Chen G, Hong Y (2022). Single-leader–multiple-followers stackelberg security game with hypergame framework. IEEE Trans. Inf. Forensics Secur..

[9] Simon, H. A. Bounded rationality. In *Utility and Probability*, 15–18 (Springer, 1990).

[10] Jiang, A. X., Nguyen, T. H., Tambe, M. & Procaccia, A. D. Monotonic maximin: A robust Stackelberg solution against boundedly rational followers. In *International Conference on Decision and Game Theory for Security*, 119–139 (Springer, 2013).

[11] Carvalho M, Ford R (2014). Moving-target defenses for computer networks. IEEE Secur. Privacy.

[12] Bondi, E. *et al.* To signal or not to signal: Exploiting uncertain real-time information in signaling games for security and sustainability. In *Proceedings of the AAAI Conference on Artificial Intelligence* 34, 1369–1377 (2020).

[13] Chen G, Ming Y, Hong Y, Yi P (2021). Distributed algorithm for $$\varepsilon$$-generalized Nash equilibria with uncertain coupled constraints. Automatica.

[14] La QD, Quek TQ, Lee J, Jin S, Zhu H (2016). Deceptive attack and defense game in honeypot-enabled networks for the internet of things. IEEE Internet Things J..

[15] Nayak, K., Kumar, S., Miller, A. & Shi, E. Stubborn mining: Generalizing selfish mining and combining with an eclipse attack. In *2016 IEEE European Symposium on Security and Privacy*, 305–320 (2016).

[16] Sanjab, A. & Saad, W. On bounded rationality in cyber-physical systems security: Game-theoretic analysis with application to smart grid protection. In *2016 Joint Workshop on Cyber-Physical Security and Resilience in Smart Grids*, 1–6 (2016).

[17] Press WH, Dyson FJ (2012). Iterated prisoner’s dilemma contains strategies that dominate any evolutionary opponent. Proc. Natl. Acad. Sci..

[18] Cheng Z, Chen G, Hong Y (2022). Misperception influence on zero-determinant strategies in iterated Prisoner’s dilemma. Sci. Rep..

[19] Wang Z, Zhou Y, Lien JW, Zheng J, Xu B (2016). Extortion can outperform generosity in the iterated Prisoner’s dilemma. Nat. Commun..

[20] Hilbe C, Nowak MA, Sigmund K (2013). Evolution of extortion in iterated Prisoner’s dilemma games. Proc. Natl. Acad. Sci..

[21] Govaert A, Cao M (2021). Zero-determinant strategies in repeated multiplayer social dilemmas with discounted payoffs. IEEE Trans. Autom. Control.

[22] Pan L, Hao D, Rong Z, Zhou T (2015). Zero-determinant strategies in iterated public goods game. Sci. Rep..

[23] Shen A, Gao Z, Gao X, Cui D (2022). The evolutionary extortion game of multiple groups in hypernetworks. Sci. Rep..

[24] Chen X, Wang L, Fu F (2022). The intricate geometry of zero-determinant strategies underlying evolutionary adaptation from extortion to generosity. New J. Phys..

[25] Taha MA, Ghoneim A (2020). Zero-determinant strategies in repeated asymmetric games. Appl. Math. Comput..

[26] McAvoy A, Hauert C (2015). Asymmetric evolutionary games. PLoS Comput. Biol..

[27] Sooksatra, K. *et al.* Solving data trading dilemma with asymmetric incomplete information using zero-determinant strategy. In *International Conference on Wireless Algorithms, Systems, and Applications*, 425–437 (Springer, 2018).

[28] Wang S, Shi H, Hu Q, Lin B, Cheng X (2019). Moving target defense for internet of things based on the zero-determinant theory. IEEE Internet Things J..

[29] Qiu, S., Wei, X., Ye, J., Wang, Z. & Yang, Z. Provably efficient fictitious play policy optimization for zero-sum Markov games with structured transitions. In *International Conference on Machine Learning*, 8715–8725 (PMLR, 2021).

[30] Guo, Q. *et al.* On the inducibility of Stackelberg equilibrium for security games. In *Proceedings of the AAAI Conference on Artificial Intelligence* 33, 2020–2028 (2019).

[31] Akin, E. The iterated Prisoner’s dilemma: Good strategies and their dynamics. *Ergodic Theory, Advances in Dynamical Systems* 77–107 (2016).

[32] Guo, Q., An, B., Bošanský, B. & Kiekintveld, C. Comparing strategic secrecy and Stackelberg commitment in security games. In *Proceedings of the 26th International Joint Conference on Artificial Intelligence*, 3691–3699 (2017).

[33] Mamiya A, Ichinose G (2020). Zero-determinant strategies under observation errors in repeated games. Phys. Rev. E.

[34] Nguyen, T. & Xu, H. Imitative attacker deception in Stackelberg security games. In *Proceedings of the 28th International Joint Conference on Artificial Intelligence*, 528–534 (2019).

[35] Li Y, Qiao S, Deng Y, Wu J (2019). Stackelberg game in critical infrastructures from a network science perspective. Phys. A Stat. Mech. Appl..

[36] Zhuang, R., DeLoach, S. A. & Ou, X. Towards a theory of moving target defense. In *Proceedings of the first ACM Workshop on Moving Target Defense*, 31–40 (2014).

[37] Shan X, Zhuang J (2018). Modeling cumulative defensive resource allocation against a strategic attacker in a multi-period multi-target sequential game. Reliab. Eng. Syst. Saf..

[38] Gal, Y. & Ghahramani, Z. Dropout as a Bayesian approximation: Representing model uncertainty in deep learning. In *International Conference on Machine Learning*, 1050–1059 (PMLR, 2016).

[39] Żychowski, A. & Mańdziuk, J. Learning attacker’s bounded rationality model in security games. In *International Conference on Neural Information Processing*, 530–539 (Springer, 2021).

[40] Chen F, Wu T, Wang L (2022). Evolutionary dynamics of zero-determinant strategies in repeated multiplayer games. J. Theor. Biol..

[41] Hilbe C, Traulsen A, Sigmund K (2015). Partners or rivals? Strategies for the iterated Prisoner’s dilemma. Games Econ. Behav..

