# Supplementary material for: Zero-determinant strategy in stochastic Stackelberg asymmetric security game
Source: Sci Rep. 2023 Jul 12;13:11308. doi: 10.1038/s41598-023-38460-8 (PMC10338512; doi:10.1038/s41598-023-38460-8)
Supplement: Supplementary file 1 — Supplementary Information. [file 41598_2023_38460_MOESM1_ESM.pdf]

Supplementary Information: Zero-Determinant Strategy in Stochastic Stackelberg Asymmetric Security Game

Zhaoyang Cheng[1,2], Guanpu Chen[3], and Yiguang Hong[4,5]

[1]Key Laboratory of Systems and Control, Academy of Mathematics and Systems Science, Beijing, 100190, China
[2]School of Mathematical Sciences, University of Chinese Academy of Sciences, Beijing, 100049, China
[3]School of Electrical Engineering and Computer Science, KTH Royal Institute of Technology, Stockholm, 11428, Sweden
[4]Department of Control Science and Engineering, Tongji University, Shanghai, 201804, China
[5]Shanghai Research Institute for Intelligent Autonomous Systems, Tongji University, Shanghai,210201, China

1 Notations

For any $\pi_d \in \Delta\mathcal{D}, \pi_a^1, \pi_a^2 \in \Delta\mathcal{A}$, and $\mathbf{f} = [f_1, f_2, f_3, f_4]^T \in \mathbb{R}^4$, denote

$$D(\pi_d,\pi_a^1,\pi_a^2,\mathbf{f}) = \begin{bmatrix} \pi_d(1|11)\pi_a^2(1|11)-1 & \pi_d(1|11)-1 & \pi_a^1(1|11)-1 & f_1 \\ \pi_d(1|12)\pi_a^2(1|12) & \pi_d(1|12)-1 & \pi_a^1(1|12) & f_2 \\ \pi_d(1|21)\pi_a^2(1|21) & \pi_d(1|21) & \pi_a^1(1|21)-1 & f_3 \\ \pi_d(1|22)\pi_a^2(1|22) & \pi_d(1|22) & \pi_a^1(1|22) & f_4 \end{bmatrix}. \tag{A.1}$$

For convenience, we take $D(\pi_d,\pi_a,\mathbf{f}) = D(\pi_d,\pi_a,\pi_a,\mathbf{f})$ and $D(\mathbf{f}) = \max_{\pi_d,\pi_a^1,\pi_a^2} D(\pi_d,\pi_a^1,\pi_a^2,\mathbf{f})$. Denote

$$J(\pi_d,\pi_a,\mathbf{f}) = D(\pi_d,\pi_a^{BR}(\pi_d),\pi_a,\mathbf{f}) + D(\pi_d,\pi_a,\pi_a^{BR}(\pi_d),\mathbf{f}),$$

and

$$C(\pi_d^{ZD},\pi_d^{SSE},\pi_a^*,\lambda) = D(\pi_d^{ZD},\lambda\pi_a^{BR}(\pi_d^{ZD})+(1-\lambda)\pi_a^*,\mathbf{1})\cdot D(\pi_d^{SSE},\lambda\pi_a^{BR}(\pi_d^{SSE})+(1-\lambda)\pi_a^*,\mathbf{1}),$$

to simplify the writing. Moreover, take

$$\begin{aligned}
A =& U_{11}^d - \frac{U_{11}^d \pi_d^{SSE}(1|21) + U_{21}^d \pi_d^{SSE}(2|11)}{\pi_d^{SSE}(2|11)+\pi_d^{SSE}(1|21)},\\
B_1 =& \max_{\pi_d^{SSE},\pi_d^{ZD}} \max_{\mathbf{f}\in\{\mathbf{1},S^d\}} \frac{1}{2}\left| D(\pi_d^{ZD},\pi_a^{BR}(\pi_d^{ZD}),S^d)J(\pi_d^{SSE},\pi_a^*,\mathbf{f}) - D(\pi_d^{SSE},\pi_a^{BR}(\pi_d^{SSE}),S^d)J(\pi_d^{ZD},\pi_a^*,\mathbf{f})\right|,\\
B_2 =& \max_{\pi_d^{SSE},\pi_d^{ZD}} \Big| D(\pi_d^{ZD},\pi_a^*,S^d)J(\pi_d^{SSE},\pi_a^*,\mathbf{1}) + D(\pi_d^{SSE},\pi_a^*,\mathbf{1})J(\pi_d^{ZD},\pi_a^*,S^d) - D(\pi_d^{SSE},\pi_a^*,S^d)J(\pi_d^{ZD},\pi_a^*,\mathbf{1})\\
&\quad - D(\pi_d^{ZD},\pi_a^*,\mathbf{1})J(\pi_d^{SSE},\pi_a^*,S^d)\Big|,\\
B_3 =& \max_{\pi_d^{SSE},\pi_d^{ZD}} \left| J(\pi_d^{ZD},\pi_a^*,S^d)J(\pi_d^{SSE},\pi_a^*,\mathbf{1}) - J(\pi_d^{ZD},\pi_a^*,S^d)J(\pi_d^{ZD},\pi_a^*,\mathbf{1})\right|,\\
B =& \max\left\{B_1, B_2, \frac{1}{2}B_3\right\}.
\end{aligned}$$

2 Proof of Lemma 1

Sufficiency: Consider that the ZD strategy π_d enforces $\eta U_d + \beta U_a + \gamma = 0$. Thus, $\pi_d(1) = \phi(\eta \mathbf{S}^d + \beta \mathbf{S}^a + \gamma) + \hat{\pi}$, where $\phi \neq 0$. Since $\pi_d \in \Xi$, the following inequalities are satisfied:

$$-1 \leqslant \phi(\eta U_i^d + \beta U_i^a + \gamma) \leqslant 0, i \in \{11, 12\}, \tag{A.2a}$$

$$0 \leqslant \phi(\eta U_j^d + \beta U_j^a + \gamma) \leqslant 1, j \in \{21, 22\}. \tag{A.2b}$$

If $\phi > 0$, it follows from the right inequalities in (A.2a) and the left inequalities in (A.2b) that

$$\begin{aligned} &\eta U_i^d + \beta U_i^a + \gamma \leqslant 0, i \in \{11,12\}, \\ &\eta U_j^d + \beta U_j^a + \gamma \geqslant 0, j \in \{21,22\}, \end{aligned} \tag{A.3}$$

which implies $\max(\eta U_{11}^d + \beta U_{11}^a, \eta U_{12}^d + \beta U_{12}^a) \leqslant -\gamma \leqslant \min(\eta U_{21}^d + \beta U_{21}^a, \eta U_{22}^d + \beta U_{22}^a)$. Similarly, we can get $\max(\eta U_{21}^d + \beta U_{21}^a, \eta U_{22}^d + \beta U_{22}^a) \leqslant -\gamma \leqslant \min(\eta U_{11}^d + \beta U_{11}^a, \eta U_{12}^d + \beta U_{12}^a)$, if $\phi < 0$.

Necessity: Consider $\max(\eta U_{11}^d + \beta U_{11}^a, \eta U_{12}^d + \beta U_{12}^a) \leqslant -\gamma \leqslant \min(\eta U_{21}^d + \beta U_{21}^d, \eta U_{22}^d + \beta U_{22}^a)$. Then we have (A.3). If all inequalities in (A.3) are not strctly satisfied, then the ZD strategy enforces $\eta U_d + \beta U_a + \gamma = 0$. Otherwise, take $\phi = \max\{|\eta U_i^d + \beta U_i^a + \gamma|\}_{i \in \{11,12,21,22\}}$, and we obtain

$$\begin{aligned} -1 \leqslant \frac{1}{\phi}(\eta U_i^d + \beta U_i^a + \gamma) \leqslant 0, i \in \{11,12\}, \\ 0 \leqslant \frac{1}{\phi}(\eta U_j^d + \beta U_j^a + \gamma) \leqslant 1, j \in \{21,22\}. \end{aligned}$$

Therefore, the ZD strategy $\pi_d^{ZD}(\frac{\eta}{\phi}, \frac{\beta}{\phi}, \frac{\gamma}{\phi})$ is feasible, and it enforces $\eta U_d + \beta U_a + \gamma = 0$. Similarly, the conclusion holds for $\max_{s \in \{21,22\}} \eta U_s^d + \beta U_s^a \leqslant -\gamma \leqslant \min_{s \in \{11,12\}} \eta U_s^d + \beta U_s^a$.

3 Proof of Theorem 1

Consider $(U_{21}^d, U_{21}^a), (U_{22}^d, U_{22}^a) \in \Gamma^+(U_{11}^d, U_{11}^a, U_{12}^d, U_{12}^a)$. Take $\eta = -\frac{U_{21}^a - U_{11}^a}{U_{21}^d - U_{11}^d}$, $\beta = 1$, $\gamma = U_{11}^d \frac{U_{21}^a - U_{11}^a}{U_{21}^d - U_{11}^d} - U_{11}^a$, and we have

$$\max(\eta U_{11}^a + \beta U_{11}^b, \eta U_{12}^a + \beta U_{12}^b) \leqslant -\gamma \leqslant \min(\eta U_{21}^a + \beta U_{21}^b, \eta U_{22}^a + \beta U_{22}^b).$$

Similarly, the conclusion holds for $(U_{21}^d, U_{21}^a), (U_{22}^d, U_{22}^a) \in \Gamma^-(U_{11}^d, U_{11}^a, U_{12}^d, U_{12}^a)$. Thus, there exists at least a ZD strategy for the defender.

4 Proof of Theorem 2

1) When $U_{11}^d \geqslant U_{21}^d$ and $U_{11}^a \geqslant U_{21}^a$, the ZD strategy $\pi^{ZD}(-k_1, 1, k_1 U_{11}^d - U_{11}^a)$ is feasible for the defender according to Lemma 1, where $0 \leqslant k_1 \leqslant \frac{U_{11}^a - U_{21}^a}{U_{11}^d - U_{21}^d}$. The ZD strategy enforces $U_a - U_{11}^a = k_1(U_d - U_{11}^d)$. Since $k_1 \geqslant 0$, the optimal utility of the attacker is U_{11}^a when the attacker observes the defender's strategy. In this case, the defender's utility is U_{11}^d. Notice that U_{11}^d is also the optimal for the defender since $U_{11}^d \leqslant \max\{U_{12}^d, U_{21}^d, U_{22}^d\}$. Then $U_d^{SSE} \leqslant U_{11}^d$. According to Lemma 2, $U_d^{SSE} = U_d(\pi_d^{ZD}, \pi_a^{BR}(\pi_d^{ZD}))$.

2) When $U_{11}^d < U_{21}^d$ and $U_{11}^a \geqslant U_{21}^a$, the ZD strategy $\pi^{ZD}(0, 1, -U_{21}^a)$ is also feasible for the defender according to Lemma 1. The ZD strategy enforces $U_a - U_{21}^a = 0$. Since the attacker always breaks ties optimally for the defender if there are multiple options, the attacker chooses the strategy which enforces $U_a = U_{21}^a$ and $U_d = \frac{(U_{21}^a - U_{11}^a)(U_{22}^d - U_{11}^d)}{U_{22}^2 - U_{11}^2} + U_{11}^d$. Thus, $U_d^{SSE} \geqslant \frac{(U_{21}^a - U_{11}^a)(U_{22}^d - U_{11}^d)}{U_{22}^2 - U_{11}^2} + U_{11}^d$. Further, suppose that the defender's utility is higher than $\frac{(U_{21}^a - U_{11}^a)(U_{22}^d - U_{11}^d)}{U_{22}^2 - U_{11}^2} + U_{11}^d$, when it chooses SSE strategy, i.e., $U_d^{SSE} > \frac{(U_{21}^a - U_{11}^a)(U_{22}^d - U_{11}^d)}{U_{22}^2 - U_{11}^2} + U_{11}^d$. Since $U_a^{SSE} - U_{11}^a \leqslant \frac{(U_{22}^a - U_{11}^a)(U_d^{SSE} - U_{11}^d)}{U_{22}^d - U_{11}^d}$ and $\frac{U_{22}^a - U_{11}^a}{U_{22}^d - U_{11}^d} < 0$, we have $U_a^{SSE} < U_{12}^a$. Actually, for any defender's strategy π_d, when the attacker chooses the strategy π_a with $\pi_a(1|11) = \pi_a(1|21) = \pi_a(2|12) = \pi_a(2|22) = 1$, $U_a(\pi_d, \pi_a) = U_{11}^a \frac{\pi_d(1|21)}{\pi_d(2|11) + \pi_d(1|21)} + U_{21}^a \frac{\pi_d(2|11)}{\pi_d(2|11) + \pi_d(1|21)} \geqslant U_{21}^a$. The attacker always has a strategy to get a utility no lower than U_{12}^a, which is conflict with $U_a^{SSE} < U_{12}^a$. Thus, $U_d^{SSE} = \frac{(U_{21}^a - U_{11}^a)(U_{22}^d - U_{11}^d)}{U_{22}^2 - U_{11}^2} + U_{11}^d = U_d(\pi_d^{ZD}, \pi_a^{BR}(\pi_d^{ZD}))$.

3) When $U_{11}^a < U_{21}^a$, the ZD strategy $\pi^{ZD}(-k_2, 1, k_2 U_{12}^d - U_{12}^a)$ is feasible for the defender according to Lemma 1, where $\frac{U_{22}^a - U_{12}^a}{U_{22}^d - U_{12}^d} \leqslant k_2 \leqslant \frac{U_{11}^a - U_{12}^a}{U_{11}^d - U_{12}^d}$. Thus, after observing the defender's strategy, the optimal utility for the attacker is U_{12}^a. In this case, the defender's corresponding utility is U_{12}^d, and $U_d(\pi_d^{ZD}, \pi_a^{BR}(\pi_d^{ZD})) - U_d(\pi_d^{SSE}, \pi_a^{BR}(\pi_d^{SSE}) = U_d^{SSE} - U_{12}^d$. Moreover, according to Lemma 1, for any ZD strategy π_d that enforces $\eta U_d + \beta U_a + \gamma = 0$, $\eta \cdot \beta > 0$ always holds when $U_{11}^a < U_{21}^a$. Thus, the attacker's BR strategy also minimizes the defender's utility, and $\max_{\pi_d^{ZD} \in \Xi} U_d(\pi_d^{ZD}, \pi_a^{BR}(\pi_d^{ZD})) = U_{12}^d$. Then $\min_{\pi_d^{ZD} \in \Xi} U_d(\pi_d^{SSE}, \pi_a^{BR}(\pi_d^{SSE})) - U_d(\pi_d^{ZD}, \pi_a^{BR}(\pi_d^{ZD})) = U_d^{SSE} - U_{12}^d$.

Therefore, $\min_{\pi_d^{ZD}\in\Xi} U_d\left(\pi_d^{SSE},\pi_a^{BR}(\pi_d^{SSE})\right)-U_d\left(\pi_d^{ZD},\pi_a^{BR}(\pi_d^{ZD})\right)=\begin{cases}0, & \text{if } U_{11}^d\geqslant U_{21}^d,\\ U_d^{SSE}-U_{12}^d, & \text{if } U_{11}^d< U_{21}^d.\end{cases}$

5 Proof of Theorem 3

According to[1], $U_d(\pi_d,\pi_a)=\frac{D(\pi_d,\pi_a,S^d)}{D(\pi_d,\pi_a,\mathbf{1})}$ and $U_a(\pi_d,\pi_a)=\frac{D(\pi_d,\pi_a,S^a)}{D(\pi_d,\pi_a,\mathbf{1})}$. For the stubborn attacker with $\pi_a^*(1|11)=\pi_a^*(1|21)=1$, we have

$$U_d(\pi_d,\pi_a^*)=U_{11}^d\frac{\pi_d(1|21)}{\pi_d(2|11)+\pi_d(1|21)}+U_{21}^d\frac{\pi_d(2|11)}{\pi_d(2|11)+\pi_d(1|21)},$$

and

$$U_a(\pi_d,\pi_a^*)=U_{11}^a\frac{\pi_d(1|21)}{\pi_d(2|11)+\pi_d(1|21)}+U_{21}^a\frac{\pi_d(2|11)}{\pi_d(2|11)+\pi_d(1|21)},$$

for any $\pi_a^*(1|s)=1$ or 0, where $s\in\{12,22\}$. Notice that $U_d(\pi_d,\pi_a^*)$ and $U_a(\pi_d,\pi_a^*)$ are monotonous in $\pi_a^*(1|s)\in[0,1]$. Then $U_d(\pi_d^{SSE},\pi_a^*)=\frac{U_{11}^d\pi_d^{SSE}(1|21)+U_{21}^d\pi_d^{SSE}(2|11)}{\pi_d^{SSE}(2|11)+\pi_d^{SSE}(1|21)}$. Take $\pi_d^{ZD}=\pi^{ZD}(-k,1,kU_{12}^d-U_{12}^a)$ with $k=\frac{U_{11}^a-U_{12}^a}{U_{11}^d-U_{12}^d}$, and we have $U_d(\pi_d^{ZD},\pi_a^*)=U_{11}^d$, which implies $U_d(\pi_d^{ZD},\pi_a^*)-U_d(\pi_d^{SSE},\pi_a^*)=U_{11}^d-\frac{U_{11}^d\pi_d^{SSE}(1|21)+U_{21}^d\pi_d^{SSE}(2|11)}{\pi_d^{SSE}(2|11)+\pi_d^{SSE}(1|21)}\geqslant 0$.

6 Proof of Theorem 4

If $U_{11}^d\geqslant U_{22}^d$ and $U_{11}^a\geqslant U_{21}^a$, the ZD strategy $\pi^{ZD}(-k_1,1,k_1U_{11}^d-U_{11}^a)$ is feasible for the defender according to Lemma 1. Moreover, according to Theorem 2, this ZD strategy is also an SSE strategy. Therefore, this ZD strategy brings the defender the same utility as the SSE strategy against a boundedly rational attacker. Thus,

$$\min_{\pi_d^{ZD}\in\Xi} U_d\left(\pi_d^{SSE},\pi_a^{\lambda}(\pi_d^{SSE},\pi_a^*)\right)-U_d\left(\pi_d^{ZD},\pi_a^{\lambda}(\pi_d^{ZD},\pi_a^*)\right)\leqslant 0,\ \text{ if }\ U_{11}^d\geqslant U_{22}^d,\ U_{11}^a\geqslant U_{21}^a.$$

Otherwise, the ZD strategy $\pi^{ZD}(-k_2,1,k_2U_{12}^d-U_{12}^a)$ is feasible for the defender by Lemma 1. According to Theorem 2, $U_d(\pi_d^{ZD},\pi_a^{BR}(\pi_d^{ZD}))-U_d(\pi_d^{SSE},\pi_a^{BR}(\pi_d^{SSE}))=-(U_d^{SSE}-U_{12}^d)$. Then

$$\frac{D(\pi_d^{ZD},\pi_a^{BR}(\pi_d^{ZD}),S^d)}{D(\pi_d^{ZD},\pi_a^{BR}(\pi_d^{ZD}),\mathbf{1})}-\frac{D(\pi_d^{SSE},\pi_a^{BR}(\pi_d^{SSE}),S^d)}{D(\pi_d^{SSE},\pi_a^{BR}(\pi_d^{SSE}),\mathbf{1})}=-(U_d^{SSE}-U_{12}^d).$$

As a result,

$$\begin{aligned}&D(\pi_d^{ZD},\pi_a^{BR}(\pi_d^{ZD}),S^d)D(\pi_d^{SSE},\pi_a^{BR}(\pi_d^{SSE}),\mathbf{1})-D(\pi_d^{SSE},\pi_a^{BR}(\pi_d^{SSE}),S^d)D(\pi_d^{ZD},\pi_a^{BR}(\pi_d^{ZD}),\mathbf{1})\\=&-(U_d^{SSE}-U_{12}^d)D(\pi_d^{ZD},\pi_a^{BR}(\pi_d^{ZD}),\mathbf{1})D(\pi_d^{SSE},\pi_a^{BR}(\pi_d^{SSE}),\mathbf{1})\end{aligned}\tag{A.4}$$

It follows from Theorem 3 that $U_d(\pi_d^{ZD},\pi_a^*)-U_d(\pi_d^{SSE},\pi_a^*)=U_{11}^a-\frac{U_{11}^a\pi_d^{SSE}(1|21)+U_{21}^a\pi_d^{SSE}(2|11)}{\pi_d^{SSE}(2|11)+\pi_d^{SSE}(1|21)}$. Similarly,

$$\frac{D(\pi_d^{ZD},\pi_a^*,S^d)}{D(\pi_d^{ZD},\pi_a^*,\mathbf{1})}-\frac{D(\pi_d^{SSE},\pi_a^*,S^d)}{D(\pi_d^{SSE},\pi_a^*,\mathbf{1})}=U_{11}^a-\frac{U_{11}^a\pi_d^{SSE}(1|21)+U_{21}^a\pi_d^{SSE}(2|11)}{\pi_d^{SSE}(2|11)+\pi_d^{SSE}(1|21)}.\tag{A.5}$$

Recall

$$U_d(\pi_d^{ZD},\lambda\pi_a^{BR}(\pi_d^{ZD})+(1-\lambda)\pi_a^*)=\frac{D(\pi_d^{ZD},\lambda\pi_a^{BR}(\pi_d^{ZD})+(1-\lambda)\pi_a^*,S^d)}{D(\pi_d^{ZD},\lambda\pi_a^{BR}(\pi_d^{ZD})+(1-\lambda)\pi_a^*,\mathbf{1})},$$

$$U_d(\pi_d^{SSE},\lambda\pi_a^{BR}(\pi_d^{SSE})+(1-\lambda)\pi_a^*)=\frac{D(\pi_d^{SSE},\lambda\pi_a^{BR}(\pi_d^{SSE})+(1-\lambda)\pi_a^*,S^d)}{D(\pi_d^{SSE},\lambda\pi_a^{BR}(\pi_d^{SSE})+(1-\lambda)\pi_a^*,\mathbf{1})}.$$

According to[1], $C(\pi_d^{ZD},\pi_d^{SSE},\pi_a^*,\lambda)\neq 0$, which was defined in 1. Without loss of generality, we consider $C(\pi_d^{ZD},\pi_d^{SSE},\pi_a^*,\lambda)>0$. As a result,

$$\begin{aligned}&U_d(\pi_d^{SSE},\lambda\pi_a^{BR}(\pi_d^{SSE})+(1-\lambda)\pi_a^*)-U_d(\pi_d^{ZD},\lambda\pi_a^{BR}(\pi_d^{ZD})+(1-\lambda)\pi_a^*)\\=&\frac{D(\pi_d^{SSE},\lambda\pi_a^{BR}(\pi_d^{SSE})+(1-\lambda)\pi_a^*,S^d)}{D(\pi_d^{SSE},\lambda\pi_a^{BR}(\pi_d^{SSE})+(1-\lambda)\pi_a^*,\mathbf{1})}-\frac{D(\pi_d^{ZD},\lambda\pi_a^{BR}(\pi_d^{ZD})+(1-\lambda)\pi_a^*,S^d)}{D(\pi_d^{ZD},\lambda\pi_a^{BR}(\pi_d^{ZD})+(1-\lambda)\pi_a^*,\mathbf{1})}\\=&\frac{1}{C(\pi_d^{ZD},\pi_d^{SSE},\pi_a^*,\lambda)}\Big(D(\pi_d^{SSE},\lambda\pi_a^{BR}(\pi_d^{SSE})+(1-\lambda)\pi_a^*,S^d)D(\pi_d^{ZD},\lambda\pi_a^{BR}(\pi_d^{ZD})+(1-\lambda)\pi_a^*,\mathbf{1})\\&-D(\pi_d^{ZD},\lambda\pi_a^{BR}(\pi_d^{ZD})+(1-\lambda)\pi_a^*,S^d)D(\pi_d^{SSE},\lambda\pi_a^{BR}(\pi_d^{SSE})+(1-\lambda)\pi_a^*,\mathbf{1})\Big).\end{aligned}$$

Actually, for any $\pi_d \in \Delta\mathcal{D}, \pi_a^1, \pi_a^2 \in \Delta\mathcal{A}, \lambda \in [0,1]$, and $\mathbf{f} = [f_1, f_2, f_3, f_4]^T \in \mathbb{R}^4$,

$$\begin{aligned}
&D(\pi_d, \lambda\pi_a^1 + (1-\lambda)\pi_a^2, \mathbf{f}) \\
=&det \begin{bmatrix} \pi_d(1|11)(\lambda\pi_a^1(1|11) + (1-\lambda)\pi_a^2(1|11)) - 1 & \pi_d(1|11) - 1 & \lambda\pi_a^1(1|11) + (1-\lambda)\pi_a^2(1|11) - 1 & f_1 \\ \pi_d(1|12)(\lambda\pi_a^1(1|12) + (1-\lambda)\pi_a^2(1|12)) & \pi_d(1|12) - 1 & \lambda\pi_a^1(1|12) + (1-\lambda)\pi_a^2(1|12) & f_2 \\ \pi_d(1|21)(\lambda\pi_a^1(1|21) + (1-\lambda)\pi_a^2(1|21)) & \pi_d(1|21) & \lambda\pi_a^1(1|21) + (1-\lambda)\pi_a^2(1|21) - 1 & f_3 \\ \pi_d(1|22)(\lambda\pi_a^1(1|22) + (1-\lambda)\pi_a^2(1|22)) & \pi_d(1|22) & \lambda\pi_a^1(1|22) + (1-\lambda)\pi_a^2(1|22) & f_4 \end{bmatrix} \\
=&det \begin{bmatrix} \pi_d(1|11)(\lambda\pi_a^1(1|11) + (1-\lambda)\pi_a^2(1|11)) - 1 & \pi_d(1|11) - 1 & \lambda\pi_a^1(1|11) - \lambda & f_1 \\ \pi_d(1|12)(\lambda\pi_a^1(1|12) + (1-\lambda)\pi_a^2(1|12)) & \pi_d(1|12) - 1 & \lambda\pi_a^1(1|12) & f_2 \\ \pi_d(1|21)(\lambda\pi_a^1(1|21) + (1-\lambda)\pi_a^2(1|21)) & \pi_d(1|21) & \lambda\pi_a^1(1|21) - \lambda & f_3 \\ \pi_d(1|22)(\lambda\pi_a^1(1|22) + (1-\lambda)\pi_a^2(1|22)) & \pi_d(1|22) & \lambda\pi_a^1(1|22) & f_4 \end{bmatrix} \\
&+ det \begin{bmatrix} \pi_d(1|11)(\lambda\pi_a^1(1|11) + (1-\lambda)\pi_a^2(1|11)) - 1 & \pi_d(1|11) - 1 & (1-\lambda)\pi_a^2(1|11) - (1-\lambda) & f_1 \\ \pi_d(1|12)(\lambda\pi_a^1(1|12) + (1-\lambda)\pi_a^2(1|12)) & \pi_d(1|12) - 1 & (1-\lambda)\pi_a^2(1|12) & f_2 \\ \pi_d(1|21)(\lambda\pi_a^1(1|21) + (1-\lambda)\pi_a^2(1|21)) & \pi_d(1|21) & (1-\lambda)\pi_a^2(1|21) - (1-\lambda) & f_3 \\ \pi_d(1|22)(\lambda\pi_a^1(1|22) + (1-\lambda)\pi_a^2(1|22)) & \pi_d(1|22) & (1-\lambda)\pi_a^2(1|22) & f_4 \end{bmatrix} \\
=&\lambda^2 D(\pi_d, \pi_a^1, \mathbf{f}) + (1-\lambda)^2 D(\pi_d, \pi_a^2, \mathbf{f}) + \lambda(1-\lambda)(D(\pi_d, \pi_a^1, \pi_a^2, \mathbf{f}) + D(\pi_d, \pi_a^2, \pi_a^1, \mathbf{f})),
\end{aligned}$$

where $D(\pi_d, \pi_a^1, \pi_a^2, \mathbf{f})$ is shown in (A.1).

Based on the above equation, we obtain

$$\begin{aligned}
&D(\pi_d^{ZD}, \lambda\pi_a^{BR}(\pi_d^{ZD}) + (1-\lambda)\pi_a^*, S^d) D(\pi_d^{SSE}, \lambda\pi_a^{BR}(\pi_d^{SSE}) + (1-\lambda)\pi_a^*, \mathbf{1}) \\
=&\Big(\lambda^2 D(\pi_d^{ZD}, \pi_a^{BR}(\pi_d^{ZD}), S^d) + (1-\lambda)^2 D(\pi_d^{ZD}, \pi_a^*, S^d) + \lambda(1-\lambda)(D(\pi_d^{ZD}, \pi_a^{BR}(\pi_d^{ZD}), \pi_a^*, S^d) \\
&+ D(\pi_d^{ZD}, \pi_a^*, \pi_a^{BR}(\pi_d^{ZD}), S^d)\Big) \times \big(\lambda^2 D(\pi_d^{SSE}, \pi_a^{BR}(\pi_d^{SSE}), \mathbf{1}) + (1-\lambda)^2 D(\pi_d^{SSE}, \pi_a^*, \mathbf{1}) \\
&+ \lambda(1-\lambda)(D(\pi_d^{SSE}, \pi_a^{BR}(\pi_d^{SSE}), \pi_a^*, \mathbf{1}) + D(\pi_d^{SSE}, \pi_a^*, \pi_a^{BR}(\pi_d^{SSE}), \mathbf{1})\big) \\
=&\lambda^4 D(\pi_d^{ZD}, \pi_a^{BR}(\pi_d^{ZD}), S^d) D(\pi_d^{SSE}, \pi_a^{BR}(\pi_d^{SSE}), \mathbf{1}) + (1-\lambda)^4 D(\pi_d^{ZD}, \pi_a^*, S^d) D(\pi_d^{SSE}, \pi_a^*, \mathbf{1}) \\
&+ \lambda^2(1-\lambda)^2 D(\pi_d^{ZD}, \pi_a^{BR}(\pi_d^{ZD}), S^d) D(\pi_d^{SSE}, \pi_a^*, \mathbf{1}) + \lambda^2(1-\lambda)^2 D(\pi_d^{SSE}, \pi_a^{BR}(\pi_d^{SSE}), S^d) D(\pi_d^{ZD}, \pi_a^*, \mathbf{1}) \\
&+ \lambda^3(1-\lambda)\Big(D(\pi_d^{ZD}, \pi_a^{BR}(\pi_d^{ZD}), S^d) J(\pi_d^{SSE}, \pi_a^*, \mathbf{1}) + D(\pi_d^{SSE}, \pi_a^{BR}(\pi_d^{SSE}), \mathbf{1}) J(\pi_d^{ZD}, \pi_a^*, S^d)\Big) \\
&+ \lambda(1-\lambda)^3\Big(D(\pi_d^{ZD}, \pi_a^*, S^d) J(\pi_d^{SSE}, \pi_a^*, \mathbf{1}) + D(\pi_d^{SSE}, \pi_a^*, \mathbf{1}) J(\pi_d^{ZD}, \pi_a^*, S^d)\Big) \\
&+ \lambda^2(1-\lambda)^2 J(\pi_d^{ZD}, \pi_a^*, S^d) J(\pi_d^{SSE}, \pi_a^*, \mathbf{1}).
\end{aligned} \tag{A.6}$$

Similarly,

$$\begin{aligned}
&D(\pi_d^{SSE}, \lambda\pi_a^{BR}(\pi_d^{SSE}) + (1-\lambda)\pi_a^*, S^d) D(\pi_d^{ZD}, \lambda\pi_a^{BR}(\pi_d^{ZD}) + (1-\lambda)\pi_a^*, \mathbf{1}) \\
=&\Big(\lambda^2 D(\pi_d^{SSE}, \pi_a^{BR}(\pi_d^{SSE}), S^d) + (1-\lambda)^2 D(\pi_d^{SSE}, \pi_a^*, S^d) + \lambda(1-\lambda)(D(\pi_d^{SSE}, \pi_a^{BR}(\pi_d^{SSE}), \pi_a^*, S^d) \\
&+ D(\pi_d^{SSE}, \pi_a^*, \pi_a^{BR}(\pi_d^{SSE}), S^d)\Big) \times \big(\lambda^2 D(\pi_d^{ZD}, \pi_a^{BR}(\pi_a^{ZD}), \mathbf{1}) + (1-\lambda)^2 D(\pi_d^{ZD}, \pi_a^*, \mathbf{1}) \\
&+ \lambda(1-\lambda)(D(\pi_d^{ZD}, \pi_a^{BR}(\pi_d^{ZD}), \pi_a^*, \mathbf{1}) + D(\pi_d^{ZD}, \pi_a^*, \pi_a^{BR}(\pi_d^{ZD}), \mathbf{1})\big) \\
=&\lambda^4 D(\pi_d^{SSE}, \pi_a^{BR}(\pi_d^{SSE}), S^d) D(\pi_d^{ZD}, \pi_a^{BR}(\pi_d^{ZD}), \mathbf{1}) + (1-\lambda)^4 D(\pi_d^{SSE}, \pi_a^*, S^d) D(\pi_d^{ZD}, \pi_a^*, \mathbf{1}) \\
&+ \lambda^2(1-\lambda)^2 D(\pi_d^{SSE}, \pi_a^{BR}(\pi_d^{SSE}), S^d) D(\pi_d^{ZD}, \pi_a^*, \mathbf{1}) + \lambda^2(1-\lambda)^2 D(\pi_d^{ZD}, \pi_a^{BR}(\pi_d^{ZD}), S^d) D(\pi_d^{SSE}, \pi_a^*, \mathbf{1}) \\
&+ \lambda^3(1-\lambda)\Big(D(\pi_d^{SSE}, \pi_a^{BR}(\pi_d^{SSE}), S^d) J(\pi_d^{ZD}, \pi_a^*, \mathbf{1}) + D(\pi_d^{ZD}, \pi_a^{BR}(\pi_d^{ZD}), \mathbf{1}) J(\pi_d^{SSE}, \pi_a^*, S^d)\Big) \\
&+ \lambda(1-\lambda)^3\Big(D(\pi_d^{SSE}, \pi_a^*, S^d) J(\pi_d^{ZD}, \pi_a^*, \mathbf{1}) + D(\pi_d^{ZD}, \pi_a^*, \mathbf{1}) J(\pi_d^{SSE}, \pi_a^*, S^d)\Big) \\
&+ \lambda^2(1-\lambda)^2 J(\pi_d^{SSE}, \pi_a^*, S^d) J(\pi_d^{ZD}, \pi_a^*, \mathbf{1}).
\end{aligned} \tag{A.7}$$

By taking the subtraction between the above two equations,

$$\begin{aligned}
&D(\pi_d^{SSE},\lambda\pi_a^{BR}(\pi_d^{SSE})+(1-\lambda)\pi_a^*,S^d)D(\pi_d^{ZD},\lambda\pi_a^{BR}(\pi_d^{ZD})+(1-\lambda)\pi_a^*,\mathbf{1})\\
&-D(\pi_d^{ZD},\lambda\pi_a^{BR}(\pi_d^{ZD})+(1-\lambda)\pi_a^*,S^d)D(\pi_d^{SSE},\lambda\pi_a^{BR}(\pi_d^{SSE})+(1-\lambda)\pi_a^*,\mathbf{1})\\
=&\lambda^4\left(D(\pi_d^{SSE},\pi_a^{BR}(\pi_d^{SSE}),S^d)D(\pi_d^{ZD},\pi_a^{BR}(\pi_d^{ZD}),\mathbf{1})-D(\pi_d^{ZD},\pi_a^{BR}(\pi_d^{ZD}),S^d)D(\pi_d^{SSE},\pi_a^{BR}(\pi_d^{SSE}),\mathbf{1})\right)\\
&+(1-\lambda)^4\left(D(\pi_d^{SSE},\pi_a^*,S^d)D(\pi_d^{ZD},\pi_a^*,\mathbf{1})-D(\pi_d^{ZD},\pi_a^*,S^d)D(\pi_d^{SSE},\pi_a^*,\mathbf{1})\right)-g,
\end{aligned}$$

where

$$\begin{aligned}
g=&\lambda^3(1-\lambda)\left(D(\pi_d^{ZD},\pi_a^{BR}(\pi_d^{ZD}),S^d)J(\pi_d^{SSE},\pi_a^*,\mathbf{1})+D(\pi_d^{SSE},\pi_a^{BR}(\pi_d^{SSE}),\mathbf{1})J(\pi_d^{ZD},\pi_a^*,S^d)\right)\\
&-\lambda^3(1-\lambda)\left(D(\pi_d^{SSE},\pi_a^{BR}(\pi_d^{SSE}),S^d)J(\pi_d^{ZD},\pi_a^*,\mathbf{1})+D(\pi_d^{ZD},\pi_a^{BR}(\pi_d^{ZD}),\mathbf{1})J(\pi_d^{SSE},\pi_a^*,S^d)\right)\\
&+\lambda(1-\lambda)^3\left(D(\pi_d^{ZD},\pi_a^*,S^d)J(\pi_d^{SSE},\pi_a^*,\mathbf{1})+D(\pi_d^{SSE},\pi_a^*,\mathbf{1})J(\pi_d^{ZD},\pi_a^*,S^d)\right)\\
&-\lambda(1-\lambda)^3\left(D(\pi_d^{SSE},\pi_a^*,S^d)J(\pi_d^{ZD},\pi_a^*,\mathbf{1})+D(\pi_d^{ZD},\pi_a^*,\mathbf{1})J(\pi_d^{SSE},\pi_a^*,S^d)\right)\\
&+\lambda^2(1-\lambda)^2J(\pi_d^{ZD},\pi_a^*,S^d)J(\pi_d^{SSE},\pi_a^*,\mathbf{1})\\
&-\lambda^2(1-\lambda)^2J(\pi_d^{SSE},\pi_a^*,S^d)J(\pi_d^{ZD},\pi_a^*,\mathbf{1}).
\end{aligned} \tag{A.8}$$

Recall the definitions of B_1, B_2, B_3, and B in 1, and we have

$$\begin{aligned}
|g|\leqslant&B\lambda^3(1-\lambda)+B\lambda(1-\lambda)^3+2B\lambda(1-\lambda)^2\\
=&B\lambda(1-\lambda)(\lambda^2+(1-\lambda)^2+2\lambda(1-\lambda))\\
=&B\lambda(1-\lambda)(\lambda+(1-\lambda))^2\\
=&B\lambda(1-\lambda).
\end{aligned} \tag{A.9}$$

Recall (A.4) and (A.5), and we obtain

$$\begin{aligned}
&D(\pi_d^{SSE},\lambda\pi_a^{BR}(\pi_d^{SSE})+(1-\lambda)\pi_a^*,S^d)D(\pi_d^{ZD},\lambda\pi_a^{BR}(\pi_d^{ZD})+(1-\lambda)\pi_a^*,\mathbf{1})\\
&-D(\pi_d^{ZD},\lambda\pi_a^{BR}(\pi_d^{ZD})+(1-\lambda)\pi_a^*,S^d)D(\pi_d^{SSE},\lambda\pi_a^{BR}(\pi_d^{SSE})+(1-\lambda)\pi_a^*,\mathbf{1})\\
\geqslant&\lambda^4\left(U_d^{SSE}-U_{12}^d\right)D(\pi_d^{ZD},\pi_a^{BR}(\pi_d^{ZD}),\mathbf{1})D(\pi_d^{SSE},\pi_a^{BR}(\pi_d^{SSE}),\mathbf{1})\\
&-(1-\lambda)^4\left(U_{11}^d-\frac{U_{11}^d\pi_d^{SSE}(1|21)+U_{21}^d\pi_d^{SSE}(2|11)}{\pi_d^{SSE}(2|11)+\pi_d^{SSE}(1|21)}\right)D(\pi_d^{ZD},\pi_a^*,\mathbf{1})D(\pi_d^{SSE},\pi_a^*,\mathbf{1})\\
&-B\lambda(1-\lambda)).
\end{aligned}$$

Actually, $D(\pi_d^{ZD},\pi_a^*,\mathbf{1})=\frac{1\cdot\pi_d^{ZD}(1|21)+1\cdot\pi_d^{ZD}(2|11)}{\pi_d^{ZD}(2|11)+\pi_d^{ZD}(1|21)}=1$, and $D(\pi_d^{SSE},\pi_a^*,\mathbf{1})=\frac{1\cdot\pi_d^{SSE}(1|21)+1\cdot\pi_d^{SSE}(2|11)}{\pi_d^{SSE}(2|11)+\pi_d^{SSE}(1|21)}=1$. Then,

$$D(\pi_d^{ZD},\pi_a^{BR}(\pi_d^{ZD}),\mathbf{1})D(\pi_d^{SSE},\pi_a^{BR}(\pi_d^{SSE}),\mathbf{1})\leqslant D(\mathbf{1}).$$

Thus, for $\lambda\in\Gamma_1$,

$$\begin{aligned}
&D(\pi_d^{SSE},\lambda\pi_a^{BR}(\pi_d^{SSE})+(1-\lambda)\pi_a^*,S^d)D(\pi_d^{ZD},\lambda\pi_a^{BR}(\pi_d^{ZD})+(1-\lambda)\pi_a^*,\mathbf{1})\\
&-D(\pi_d^{ZD},\lambda\pi_a^{BR}(\pi_d^{ZD})+(1-\lambda)\pi_a^*,S^d)D(\pi_d^{SSE},\lambda\pi_a^{BR}(\pi_d^{SSE})+(1-\lambda)\pi_a^*,\mathbf{1})\\
\geqslant&\lambda^4\left(U_d^{SSE}-U_{12}^d\right)D(\mathbf{1})-A(1-\lambda)^4-B\lambda(1-\lambda).
\end{aligned}$$

For any $\lambda\in\Gamma_1=\{\lambda\in[0,1]|(U_d^{SSE}-U_{12}^d)D(\mathbf{1})\lambda^4-A(1-\lambda)^4-B\lambda(1-\lambda)\geqslant 0\}$, we have

$$U_d\left(\pi_d^{SSE},\pi_d^{\lambda}(\pi_d^{SSE},\pi_d^*)\right)\geqslant U_d\left(\pi_d^{ZD},\pi_d^{\lambda}(\pi_d^{ZD},\pi_d^*)\right).$$

Moreover,

$$
\begin{aligned}
&U_d(\pi_d^{SSE},\lambda\pi_a^{BR}(\pi_d^{SSE})+(1-\lambda)\pi_a^*)-U_d(\pi_d^{ZD},\lambda\pi_a^{BR}(\pi_d^{ZD})+(1-\lambda)\pi_a^*)\\
=&\frac{D(\pi_d^{SSE},\lambda\pi_a^{BR}(\pi_d^{SSE})+(1-\lambda)\pi_a^*,S^d)}{D(\pi_d^{SSE},\lambda\pi_a^{BR}(\pi_d^{SSE})+(1-\lambda)\pi_a^*,\mathbf{1})}-\frac{D(\pi_d^{ZD},\lambda\pi_a^{BR}(\pi_d^{ZD})+(1-\lambda)\pi_a^*,S^d)}{D(\pi_d^{ZD},\lambda\pi_a^{BR}(\pi_d^{ZD})+(1-\lambda)\pi_a^*,\mathbf{1})}\\
=&\frac{1}{C(\pi_d^{ZD},\pi_d^{SSE},\pi_a^*,\lambda)}\Big(D(\pi_d^{SSE},\lambda\pi_a^{BR}(\pi_d^{SSE})+(1-\lambda)\pi_a^*,S^d)D(\pi_d^{ZD},\lambda\pi_a^{BR}(\pi_d^{ZD})+(1-\lambda)\pi_a^*,\mathbf{1})\\
&-D(\pi_d^{ZD},\lambda\pi_a^{BR}(\pi_d^{ZD})+(1-\lambda)\pi_a^*,S^d)D(\pi_d^{SSE},\lambda\pi_a^{BR}(\pi_d^{SSE})+(1-\lambda)\pi_a^*,\mathbf{1})\Big)\\
\leqslant&\frac{\left(U_d^{SSE}-U_{12}^d\right)C(\pi_d^{ZD},\pi_d^{SSE},\pi_a^*,1)\lambda^4-A(1-\lambda)^4+B\lambda(1-\lambda)}{C(\pi_d^{ZD},\pi_d^{SSE},\pi_a^*,\lambda)}\\
=&H(\pi_d^{ZD},\pi_d^{SSE},\pi_a^*,\lambda),
\end{aligned}
$$

where $H(\pi_d^{ZD},\pi_d^{SSE},\pi_a^*,\lambda)$ was defined in (5). Therefore,

$$
\min_{\pi_d^{ZD}\in\Xi} U_d\left(\pi_d^{SSE},\pi_a^\lambda(\pi_d^{SSE},\pi_a^*)\right)-U_d\left(\pi_d^{ZD},\pi_a^\lambda(\pi_d^{ZD},\pi_a^*)\right)\leqslant\begin{cases}0, & \text{if } U_{11}^d\geqslant U_{22}^d,\ U_{11}^a\geqslant U_{21}^a,\\ H(\pi_d^{ZD},\pi_d^{SSE},\pi_a^*,\lambda), & \text{otherwise.}\end{cases}
$$

7 Proof of Theorem 5

The ZD strategy $\pi_d^{ZD}=\pi^{ZD}(-k,1,kU_{21}^d-U_{21}^a)$ is feasible for the defender according to Lemma 1. Similar to the analysis in the proof of Theorem 4, we also obtain (A.4) and (A.5). Then

$$
\begin{aligned}
&\Big(D(\pi_d^{ZD},\lambda\pi_a^{BR}(\pi_d^{ZD})+(1-\lambda)\pi_a^*,S^d)D(\pi_d^{SSE},\lambda\pi_a^{BR}(\pi_d^{SSE})+(1-\lambda)\pi_a^*,\mathbf{1})\\
&-D(\pi_d^{SSE},\lambda\pi_a^{BR}(\pi_d^{SSE})+(1-\lambda)\pi_a^*,S^d)D(\pi_d^{ZD},\lambda\pi_a^{BR}(\pi_d^{ZD})+(1-\lambda)\pi_a^*,\mathbf{1})\Big)\\
=&\lambda^4\left(\pi_d^{ZD},\pi_a^{BR}(\pi_d^{ZD}),S^d)D(\pi_d^{SSE},\pi_a^{BR}(\pi_d^{SSE}),\mathbf{1})-D(\pi_d^{SSE},\pi_a^{BR}(\pi_d^{SSE}),S^d)D(\pi_d^{ZD},\pi_a^{BR}(\pi_d^{ZD}),\mathbf{1})\right)\\
&+(1-\lambda)^4\left(D(\pi_d^{ZD},\pi_a^*,S^d)D(\pi_d^{SSE},\pi_a^*,\mathbf{1})-D(\pi_d^{SSE},\pi_a^*,S^d)D(\pi_d^{ZD},\pi_a^*,\mathbf{1})\right)+g,
\end{aligned}
$$

where g is shown in (A.8). Since $|g|\leqslant B\lambda(1-\lambda)$ according to (A.9), we have

$$
\begin{aligned}
&D(\pi_d^{ZD},\lambda\pi_a^{BR}(\pi_d^{ZD})+(1-\lambda)\pi_a^*,S^d)D(\pi_d^{SSE},\lambda\pi_a^{BR}(\pi_d^{SSE})+(1-\lambda)\pi_a^*,\mathbf{1})\\
&-D(\pi_d^{SSE},\lambda\pi_a^{BR}(\pi_d^{SSE})+(1-\lambda)\pi_a^*,S^d)D(\pi_d^{ZD},\lambda\pi_a^{BR}(\pi_d^{ZD})+(1-\lambda)\pi_a^*,\mathbf{1})\\
\geqslant&\lambda^4\left(U_{12}^d-U_d^{SSE}\right)D(\pi_d^{ZD},\pi_a^{BR}(\pi_d^{ZD}),\mathbf{1})D(\pi_d^{SSE},\pi_a^{BR}(\pi_d^{SSE}),\mathbf{1})\\
&+(1-\lambda)^4\left(U_{11}^d-\frac{U_{11}^d\pi_d^{SSE}(1|21)+U_{21}^d\pi_d^{SSE}(2|11)}{\pi_d^{SSE}(2|11)+\pi_d^{SSE}(1|21)}\right)D(\pi_d^{ZD},\pi_a^*,\mathbf{1})D(\pi_d^{SSE},\pi_a^*,\mathbf{1})\\
&-B\lambda(1-\lambda))\\
\geqslant&\lambda^4\left(U_{12}^d-U_d^{SSE}\right)D(\mathbf{1})+A(1-\lambda)^4-B\lambda(1-\lambda).
\end{aligned}
$$

As a result,

$$
\begin{aligned}
&U_d(\pi_d^{ZD},\lambda\pi_a^{BR}(\pi_d^{ZD})+(1-\lambda)\pi_a^*)-U_d(\pi_d^{SSE},\lambda\pi_a^{BR}(\pi_d^{SSE})+(1-\lambda)\pi_a^*)\\
=&\frac{D(\pi_d^{ZD},\lambda\pi_a^{BR}(\pi_d^{ZD})+(1-\lambda)\pi_a^*,S^d)}{D(\pi_d^{ZD},\lambda\pi_a^{BR}(\pi_d^{ZD})+(1-\lambda)\pi_a^*,\mathbf{1})}-\frac{D(\pi_d^{SSE},\lambda\pi_a^{BR}(\pi_d^{SSE})+(1-\lambda)\pi_a^*,S^d)}{D(\pi_d^{SSE},\lambda\pi_a^{BR}(\pi_d^{SSE})+(1-\lambda)\pi_a^*,\mathbf{1})}\\
=&\frac{1}{C(\pi_d^{ZD},\pi_d^{SSE},\pi_a^*,\lambda)}\Big(D(\pi_d^{ZD},\lambda\pi_a^{BR}(\pi_d^{ZD})+(1-\lambda)\pi_a^*,S^d)D(\pi_d^{SSE},\lambda\pi_a^{BR}(\pi_d^{SSE})+(1-\lambda)\pi_a^*,\mathbf{1})\\
&-D(\pi_d^{SSE},\lambda\pi_a^{BR}(\pi_d^{SSE})+(1-\lambda)\pi_a^*,S^d)D(\pi_d^{ZD},\lambda\pi_a^{BR}(\pi_d^{ZD})+(1-\lambda)\pi_a^*,\mathbf{1})\Big).
\end{aligned}
$$

Thus,

$$
U_d(\pi_d^{ZD},\lambda\pi_a^{BR}(\pi_d^{ZD})+(1-\lambda)\pi_a^*)\geqslant U_d(\pi_d^{SSE},\lambda\pi_a^{BR}(\pi_d^{SSE})+(1-\lambda)\pi_a^*),
$$

which implies the conclusion.

8 Algorithms

We show the details of the mentioned algorithms in Applications. Here, we utilize the fictitious play method[2] and the Q-learning method[3] for the BR strategy of the attacker according to players' action history.

Algorithm A.1 Fictitious Play of the Boundedly Rational Attacker

Input: Rational factor: λ, stubborn strategy: π_a^*.
Initialize: The defender's strategy frequency $\hat{\pi}_d(d|s) = 0$ for all $d \in \mathcal{D}, s \in \mathcal{S}$, and its average payoff $U_d = 0$.

for $t = 1, 2, \cdots$ **do**
  The defender takes $d_t \sim \pi_d$.
  The attacker takes $a_t \sim \pi_a$.
  Reach the state $s(t)$, and players get the payoff $r_d(d_t, a_t)$ and $r_a(d_t, a_t)$.
  $\hat{\pi}_d(d_t|s_{t-1}) = \frac{(t-1)\hat{\pi}_d(d_t|s_{t-1})+1}{t}$.
  $\pi_a = \lambda BR(\hat{\pi}_d) + (1-\lambda)\pi_a^*$.
  $U_d = \sum_{i=1}^{t} \frac{r_d(d_i, a_i)}{t}$.
end for

Algorithm A.2 Q-learning of the Boundedly Rational Attacker

Input: Rational factor: λ, stubborn strategy: π_a^*, ε_1, ε_2.
Initialize: $Q(s,a) = 0$ for $s \in \mathcal{S}, a \in \mathcal{A}$, $\bar{r}_a = 0$, and $U_d = 0$.

for $t = 1, 2, \cdots$ **do**
  The defender takes $d_t \sim \pi_d$.
  The attacker takes a_t with $(1-\lambda)$-greedy strategy based on $Q(s(t-1), b)$.
  Reach the state $s(t)$. Get $r_d(d_t, a_t)$ and $r_a(d_t, a_t)$.
  $\delta = r_a(d_t, a_t) - \bar{r}_a + \max_{a'} Q(s(t), a') - Q(s(t-1), a_t)$.
  $Q(s(t-1), a_t) = Q(s(t-1), a_t) + \varepsilon_1 \delta$.
  if $Q(s(t-1), a_t) = \max_{b'} Q(s(t-1), a)$ **then**
    $\bar{r}_a = (1-\varepsilon_2)\bar{r}_a + \varepsilon_2 \frac{(t-1)\bar{r}_a + r_a(d_t, a_t)}{t}$.
  end if
  $U_d = \sum_{i=1}^{t} \frac{r_d(d_i, a_i)}{t}$.
end for

References

1. Press, W. H. & Dyson, F. J. Iterated prisoner's dilemma contains strategies that dominate any evolutionary opponent. *Proc. Natl. Acad. Sci.* **109**, 10409–10413 (2012).
2. Qiu, S., Wei, X., Ye, J., Wang, Z. & Yang, Z. Provably efficient fictitious play policy optimization for zero-sum markov games with structured transitions. In *International Conference on Machine Learning*, 8715–8725 (PMLR, 2021).
3. Li, K. & Hao, D. Cooperation enforcement and collusion resistance in repeated public goods games. In *Proceedings of the AAAI Conference on Artificial Intelligence*, vol. 33, 2085–2092 (2019).
